# Brigatinib combined with anti-EGFR antibody overcomes osimertinib resistance in EGFR-mutated non-small-cell lung cancer

**DOI:** 10.1038/ncomms14768

**Published:** 2017-03-13

**Authors:** Ken Uchibori, Naohiko Inase, Mitsugu Araki, Mayumi Kamada, Shigeo Sato, Yasushi Okuno, Naoya Fujita, Ryohei Katayama

**Affiliations:** 1 Cancer Chemotherapy Center, Japanese Foundation for Cancer Research, 3-8-31, Ariake, Koto-ku, Tokyo 135-8550, Japan; 2 Department of Respiratory Medicine, Graduate School of Medical and Dental Sciences, Tokyo Medical and Dental University, 1-5-45 Yushima, Bunkyo-ku, Tokyo 113-8510, Japan; 3 RIKEN Advanced Institute for Computational Science, 7-1-26 Minatojima-Minamimachi, Chuo-ku, Kobe, Hyogo 650-0047, Japan; 4 Graduate School of Medicine, Kyoto University, 54 Shogoin-Kawaharacho, Sakyo-ku, Kyoto 606-8507, Japan

## Abstract

Osimertinib has been demonstrated to overcome the epidermal growth factor receptor (EGFR)-T790M, the most relevant acquired resistance to first-generation EGFR–tyrosine kinase inhibitors (EGFR–TKIs). However, the C797S mutation, which impairs the covalent binding between the cysteine residue at position 797 of EGFR and osimertinib, induces resistance to osimertinib. Currently, there are no effective therapeutic strategies to overcome the C797S/T790M/activating-mutation (triple-mutation)-mediated EGFR–TKI resistance. In the present study, we identify brigatinib to be effective against triple-mutation-harbouring cells *in vitro* and *in vivo*. Our original computational simulation demonstrates that brigatinib fits into the ATP-binding pocket of triple-mutant EGFR. The structure–activity relationship analysis reveals the key component in brigatinib to inhibit the triple-mutant EGFR. The efficacy of brigatinib is enhanced markedly by combination with anti-EGFR antibody because of the decrease of surface and total EGFR expression. Thus, the combination therapy of brigatinib with anti-EGFR antibody is a powerful candidate to overcome triple-mutant EGFR.

## Abstract (web summary)

Resistance to EGFR-tyrosine kinase inhibitors in non-small-cell lung cancer is mediated by the C797S/T790M/activating mutation. Here, the authors identify brigatinib as a potential drug that in combination with anti-EGFR can overcome resistance in mutated cells.

Non-small-cell lung cancer (NSCLC) harbouring an epidermal growth factor receptor (EGFR)-activating mutation accounts for ∼30–40% of NSCLC in the Japanese population and ∼15% in Caucasians^1^. For the treatment of EGFR-mutated NSCLC, EGFR–tyrosine kinase inhibitors (EGFR–TKIs) that inhibit the EGFR-induced downstream signalling pathway by binding to the ATP-binding pocket of the EGFR–tyrosine kinase domain have been evaluated and are currently being clinically used^2^,^3^,^4^,^5^,^6^,^7^,^8^,^9^. The use of EGFR–TKIs has improved prognoses in patients with EGFR-mutated lung cancer^10^.

Before clinical application of EGFR–TKIs, the overall survival of NSCLC patients was only approximately a year, as shown in the trials verifying the efficacy of platinum doublets chemotherapy in metastatic NSCLC^11^. Several clinical trials of EGFR–TKIs in EGFR-activating mutation-positive NSCLC patients have shown improved survival of 2 to nearly 3 years^12^,^13^,^14^. The importance of treatment with the appropriate molecularly targeted drugs in driver oncogene-positive NSCLC has been increasingly recognized. Kris *et al*.^15^ reported the benefit of precisely identifying the target oncogenes in cancer cells and providing the appropriate molecularly targeted therapy.

Although the benefits of molecularly targeted drugs are substantial, most patients experience a recurrence of disease within ∼1–2 years due to acquired resistance. The acquired resistance against the first-generation EGFR–TKIs gefitinib and erlotinib has been revealed to be mainly caused by a gatekeeper mutation^16^,^17^ involving substitution of threonine at position 790 with methionine (T790M), which hampers the binding of the EGFR–TKI to the ATP-binding site of EGFR and accounts for 60–70% of resistant cases^18^,^19^. Many clinical trials of various agents to overcome the acquired resistance to gefitinib or erlotinib were tried but failed to show any clinical advantage, except for one trial in which afatinib+cetuximab achieved a response rate of 29% (refs 20, 21, 22, 23, 24, 25). Despite its efficacy, this combination treatment has not been used in the clinical setting because of its relatively severe toxicity. To resolve this difficult situation, covalently binding third-generation EGFR–TKIs selectively targeting T790M have been evaluated for the treatment of patients with advanced EGFR-mutated NSCLC^26^,^27^,^28^. Osimertinib, which was reported to be efficacious in T790M mutation-positive EGFR-mutated NSCLC^29^, has been approved in the US and other countries. Jänne *et al*.^30^ reported that in a phase 1/2 trial of osimertinib, among 127 patients with confirmed EGFR T790M who could be evaluated, the response rate was 61% and the median progression-free survival was 9.6 months, which is as long as that of first-line EGFR–TKIs for EGFR-mutated lung cancer.

Approval of osimertinib will influence the treatment tactics for EGFR-mutated lung cancer, but, again, resistance to osimertinib will be a major obstacle. In 2015, various mechanisms of the acquired resistance against osimertinib were independently published by several groups. An EGFR mutation involving substitution of cysteine at position 797 with serine (C797S) was detected in cell-free plasma DNA from osimertinib-refractory patients and was shown to induce osimertinib resistance^31^. Ercan *et al*.^32^ reported that Ba/F3 cells with three amino acid substitutions, L844V, L718Q and C797S, found using the *N*-ethyl-*N*-nitrosourea mutagenesis method, are totally refractory to the third-generation EGFR–TKIs WZ-4002, osimertinib and CO-1686. In cases without the C797S mutation, although loss of T790M was reported as specific resistant mechanism to third-generation EGFR–TKIs, bypass pathway activation, such as c-MET activation or small-cell lung cancer (SCLC) transformation, in resistant tumours is thought to be the mechanism of resistance similar to that known in first-generation EGFR–TKIs^33^,^34^,^35^,^36^,^37^,^38^,^39^. Indeed, *HER2* amplification, *Met* amplification, *BRAF* mutation and SCLC transformation have been observed in osimertinib-resistant cases^40^,^41^. Therefore, new therapeutic strategies are needed to overcome the resistance to the third-generation EGFR–TKIs.

The osimertinib resistance due to the loss of T790M or bypass pathway activation is expected to be overcome using existing methods, for example, exchange to or addition of a first-generation EGFR–TKI or concurrent combination therapy of an inhibiting alternative pathway, respectively. However, we now have no clinically available strategy to conquer the C797S/T790M/activating-mutation (triple-mutation). Recently, Jia *et al*.^42^ published a unique allosteric EGFR inhibitor that can overcome the EGFR–TKI resistance by EGFR-C797S/T790M/L858R mutation, but not EGFR-C797S/T790M/del19-mediated resistance by treating in combination with cetuximab. Niederst *et al*.^43^ investigated the emergence of the C797S allele and found that if C797S developed *in trans* of the T790M allele, a combination of first- and third-generation EGFR–TKIs may be effective enough for clinical use; however, when the C797S and T790M mutations developed *in cis*, all sensitivity to any of the existing EGFR–TKIs, including the third-generation ones, was lost. However, we currently have no information on whether the *in vitro* efficacy of the combination of first- and third-generation EGFR–TKIs for *trans* C797S is clinically reproducible. The C797S mutations found in the samples obtained from participants in the osimertinib trial mentioned above were all *in cis* alleles except for one case of *in trans*^31^. The frequency of resistance caused by C797S emergence is not well known because of the small number of third-generation-resistant patients, but the importance of developing treatment strategies for this group will be increasing in the near future as more and more patients with EGFR-mutated NSCLC will be receiving osimertinib.

To investigate the therapeutic strategies for treating patients with triple-mutant EGFR, we performed drug screening and found brigatinib to be the promising candidate against triple-mutant EGFR with less potency against wild-type EGFR according to the *in vitro* and *in vivo* assays. Structure–activity relationship analysis and computational simulation reveal the key component determining the affinity and the binding mode to triple-mutant EGFR that are expected to attribute to the future development. Finally, the combination with anti-EGFR antibody strikingly reduces the IC_50_ of brigatinib and prolongs the survival of the triple-mutant EGFR xenograft-bearing mice. These findings in this study may help overcome acquired resistance to third-generation EGFR–TKIs.

## Results

### Drug resistance by EGFR-C797S/T790M/activating mutations

Currently, there are four EGFR–TKIs available in the clinical setting—gefitinib, erlotinib, afatinib and osimertinib. Gefitinib and erlotinib are so-called first-generation EGFR–TKIs that were proven to be efficacious for NSCLC harbouring an EGFR mutation (EGFR-activating mutation; exon 19 deletion [del19] or L858R point mutation in exon 21 [L858R]). Afatinib is a second-generation EGFR–TKI irreversibly targeting the pan-HER signal pathway. Osimertinib and EGF-816 are third-generation EGFR–TKIs that covalently bind to EGFR and are effective against the T790M-mutated EGFR, the most common mechanism of acquired resistance to first-generation EGFR–TKIs. EGF-816 is not yet accessible except for clinical trials. All classes of EGFR–TKIs are active against the EGFR-activating mutation alone. Therefore, we evaluated the sensitivity of the EGFR–TKI-resistant mutations introduced into Ba/F3 cells (T790M/activating mutation or C797S/T790M/activating mutation (triple-mutation)) to the clinically relevant EGFR–TKIs gefitinib, afatinib, osimertinib and EGF-816.

The CellTiter-Glo assay showed that gefitinib and afatinib were effective against the EGFR-activating mutation, as previously described, and also potent against the double mutation with C797S, which is the covalent binding site of the second- and third-generation EGFR–TKIs (Supplementary Fig. 1a–d). However, they are no longer effective against the T790M gatekeeper mutation, the most relevant mechanism of resistance to the first-generation EGFR–TKIs. Osimertinib and EGF-816 showed efficacy not only against the EGFR-activating mutation alone but also against the double mutation with T790M *in vitro* (Supplementary Fig. 1e,f). Although the resistance due to the T790M mutation has been shown to be overcome by the third-generation EGFR–TKIs, they lost their inhibitory activity when the C797S mutation occurred concurrent with the T790M *in cis*. The Ba/F3 cells expressing the triple-mutant EGFR were entirely resistant to all generations of EGFR–TKIs with similar IC_50_ values as in the parental Ba/F3 cells (Supplementary Fig. 1g–i and Table 1a,b). We examined the sensitivity of PC9 cells (parental; expressing del19 alone) and resistant PC9 cells (T790M; double mutation del19 with T790M, triple-mutant; generated by introducing C797S/T790M/del19 (triple-del19) mutant EGFR) to the EGFR–TKIs to confirm the characteristics demonstrated in Ba/F3 cells. The CellTiter-Glo assay revealed similar results in the PC9 triple-mutant cells that were also refractory to all EGFR–TKIs as seen in Ba/F3 cells (Supplementary Fig. 2a–c and Table 1c). Interestingly, PC9 triple-mutant cells showed no sensitivity to the combination therapy of gefitinib and osimertinib that was shown to overcome the acquired osimertinib resistance mediated by C797S and T790M mutations *in trans* (Supplementary Fig. 2d). These results suggest that no clinically beneficial drug is available for the treatment of the triple-mutant EGFR.

### Brigatinib overcomes the resistance of EGFR-triple-mutant

**Figure 1:**
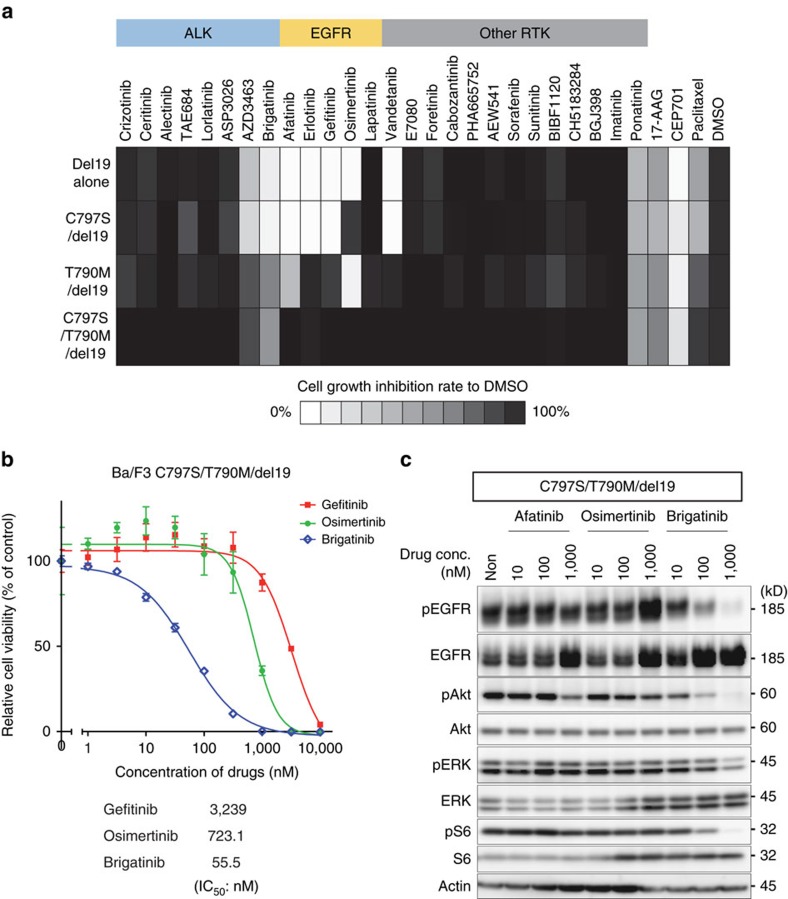
Identification of brigatinib as an EGFR-C797S/T790M/activating-mutation (triple-mutant EGFR) inhibitor. (**a**) The results of screening the growth-inhibitory activity of 30 drugs in Ba/F3 cells expressing four types of EGFR-del19 with or without T790M or C797S mutations are shown in a heat map. Ba/F3 cells expressing each EGFR mutant were treated with 100 nM of the indicated inhibitors. After 72 h of drug treatment, the cell viability was measured using the CellTiter-Glo assay. Relative cell viability was calculated from each value divided by the DMSO control. Among the inhibitors, only brigatinib and ponatinib were sufficiently efficacious against the triple-mutant EGFR. AZD3463 acted as a weak inhibitor to the triple mutation. (**b**) Growth inhibition assessed by the CellTiter-Glo assay of EGFR-C797S/T790M/del19 (triple-del19)-mutated Ba/F3 cells treated with gefitinib, osimertinib and brigatinib.; *N*=3. Results are expressed as mean±s.d. IC_50_ values were calculated using growth inhibition assay. (**c**) Phosphorylation of EGFR and downstream signals were significantly inhibited by brigatinib in Ba/F3 cells expressing triple-del19 even though afatinib and osimertinib did not suppress at all the EGFR signalling of triple-del19.

To investigate the candidates who could overcome the triple-mutant EGFR, we performed a focused drug screening to examine their efficacy against each type of EGFR-del19 mutation in Ba/F3 cells using the CellTiter-Glo assay. The 30 drugs used in the focused drug screening comprised not only EGFR–TKIs but also kinase inhibitors targeting other tyrosine kinases or serine/threonine kinases that are now available clinically or are being evaluated in clinical trials, referring to the report by Duong-Ly *et al*.^44^ that showed the potential to repurpose inhibitors against disease-associated or drug-resistant mutant kinases. Among TKIs, only brigatinib and ponatinib were expected to have inhibitory activity against EGFR-triple-del19 with ∼50% growth inhibition at 100 nM (Fig. 1a). However, the potency of ponatinib against triple-del19 assessed by the CellTiter-Glo assay was disappointing with almost the same IC_50_ value as that in the parental Ba/F3 cells (Supplementary Fig. 3). We then evaluated the efficacy of brigatinib in T790M/del19 and triple-del19-mutated EGFR-expressing Ba/F3 cells using the CellTiter-Glo assay compared with other clinically available EGFR–TKIs. The assay demonstrated that only brigatinib inhibited the growth of Ba/F3 cells expressing the triple-del19 mutation with a low IC_50_ (<100 nM; Fig. 1b). The addition of IL-3 as an original survival signalling pathway activator counteracted this inhibitory effect of brigatinib. The potency of brigatinib in triple-del19 was confirmed by western blotting that showed decreased phosphorylation of EGFR and its downstream signalling pathway in a dose-dependent manner in contrast to the lack of inhibition of EGFR phosphorylation by afatinib and osimertinib (Fig. 1c). Although brigatinib showed acceptable inhibitory activity in cell growth inhibition and EGFR signal pathway also in Ba/F3 cells expressing EGFR-del19 alone, T790M/del19 or C797S/del19, its efficacy was only superior to that of osimertinib against C797S/del19 (Supplementary Fig. 4). The cell growth inhibition assay and western blotting using same setting of EGFR–TKIs in Ba/F3 cells expressing mutant EGFR activated by L858R (L858R alone, T790M/L858R, C797S/L858R and C797S/T790M/L858R (triple-L858R)) showed that brigatinib was also effective in EGFR-L858R series but was less potent than in del19, with similar pattern of efficacy observed in Ba/F3 cells expressing corresponding mutation types of EGFR activated by del19 (Supplementary Fig. 5).

**Figure 2:**
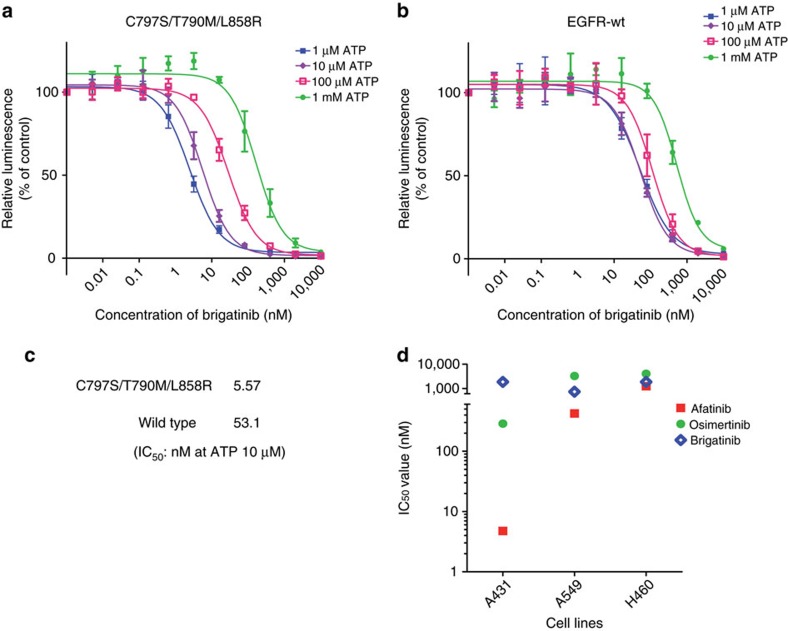
Brigatinib inhibited EGFR through ATP competition and was less potent to wild-type EGFR or non-EGFR-mutated cells. (**a**–**c**) The evaluation of the inhibitory activity of brigatinib in the *in vitro* kinase assay using the ADP-Glo assay kit showed a dose-dependent decrease in EGFR activity with brigatinib according to the increase of ATP concentration in either (**a**) EGFR-C797S/T790M/L858R or (**b**) wild type; *N*=3. Results are expressed as mean±s.d. (**c**) IC_50_ value calculated at an ATP concentration of 10 μM suggested the better affinity of brigatinib to EGFR-C797S/T790M/L858R than to wild-type EGFR. (**d**) IC_50_ values calculated from the cell viability assay of non-EGFR-mutated cell lines, A431, A549 and H460, assessed using CellTiter-Glo assay kit are shown with a dot plot.

To evaluate whether or not brigatinib acts on EGFR through ATP competition, and to compare its activity between triple-mutant EGFR and wild-type EGFR, an *in vitro* kinase assay was performed using an ADP-Glo kit. The kinase activity inhibition curves demonstrated by this assay shifted with the ATP concentrations in both the triple-mutant and wild-type EGFR, indicating that brigatinib competitively affected the ATP-binding site of the EGFR kinase domain (Fig. 2a,b). The higher potency of brigatinib to triple-mutant EGFR was confirmed by the IC_50_ value calculated for 10 μM ATP, which was ∼10 times lower for triple-L858R than for the wild type (Fig. 2c). Furthermore, brigatinib showed less inhibitory activity to the cell lines without EGFR mutation than afatinib and osimertinib when compared with the IC_50_ values of each drugs, especially in the wild-type EGFR-amplified A431 cells. In the KRAS-mutated A549 or H460 cells, all these inhibitors had high IC_50_ values. From these results, brigatinib was expected to have a preferable toxicity profile related to wild-type EGFR inhibition compared with afatinib or even osimertinib (Fig. 2d and Supplementary Fig. 6a–c).

### The activity of other ALK inhibitors with similar structure

**Figure 3:**
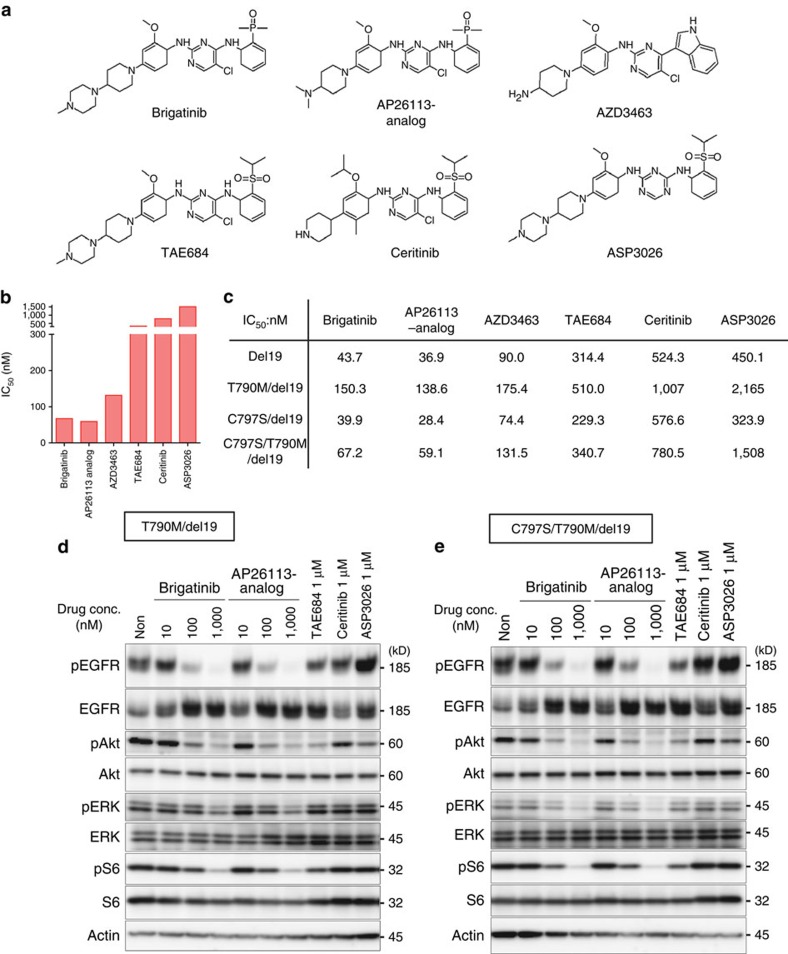
Efficacy of brigatinib and similarly structured drugs in the EGFR-mutated Ba/F3 cells and their chemical structures. (**a**) Chemical structures of six ALK–TKIs were very similar. (**b**,**c**) IC_50_ values in Ba/F3 cells expressing four mutation types of EGFR-del19 were obtained by treatment with brigatinib, AP26113-analog, AZD3463, TAE684, ceritinib and ASP3026 for 72 h. Those of C797S/T790M/del19 were shown by bar graph (**b**) and those of all mutation types were demonstrated by a table (**c**). The CellTiter-Glo assay was used to measure cell viability. (**d**,**e**) Ba/F3 cells expressing T790M/del19 (**d**) or C797S/T790M/del19 (**e**) were treated with the indicated concentrations of brigatinib, AP26113 analog, TAE684, ceritinib or ASP3026 for 6 h. Phosphorylation of EGFR and its downstream signals were evaluated by western blotting with the indicated antibodies.

As brigatinib was developed as a next-generation anaplastic lymphoma kinase (ALK)–TKI^45^,^46^, we then investigated several ALK–TKIs with similar structures—AP26113-analog, AZD3463, TAE684, ceritinib and ASP3026 (Fig. 3a). Cell growth inhibition experiments using the CellTiter-Glo assay demonstrated that brigatinib and AP26113-analog had similar potency against the triple mutation with IC_50_ values of <100 nM, whereas AZD3463 was moderately active followed by TAE684 and two other drugs that showed no activity (Fig. 3b,c and Supplementary Fig. 7a–d). The differences in efficacy between these drugs in cell growth inhibition were reproduced in western blotting experiments showing that brigatinib and AP26113-analog more effectively suppressed phosphorylation of EGFR and its downstream signalling pathway in cells expressing all types of EGFR mutations (Fig. 3d,e and Supplementary Fig. 7e,f). These experiments also suggested that L858R tends to be less sensitive to inhibitors than del19 (Supplementary Figs 8,9). Comparison of the chemical structures of the ALK–TKIs suggested that some functional groups have a key role in increasing the binding affinity to the triple-mutant EGFR. First, the phosphine oxide group in brigatinib and AP26113-analog, but not the isopropylsulfonyl group in TAE684, ceritinib and ASP3026, might contribute to their greater activity against the triple-mutant EGFR. Second, the presence of an isopropoxy group in ceritinib but no methoxy group in other ALK–TKIs and the absence of chloride in ASP3026 might work as negative factors (Fig. 3).

### *In silico* simulation of brigatinib binds to triple-mutant EGFR

**Figure 4:**
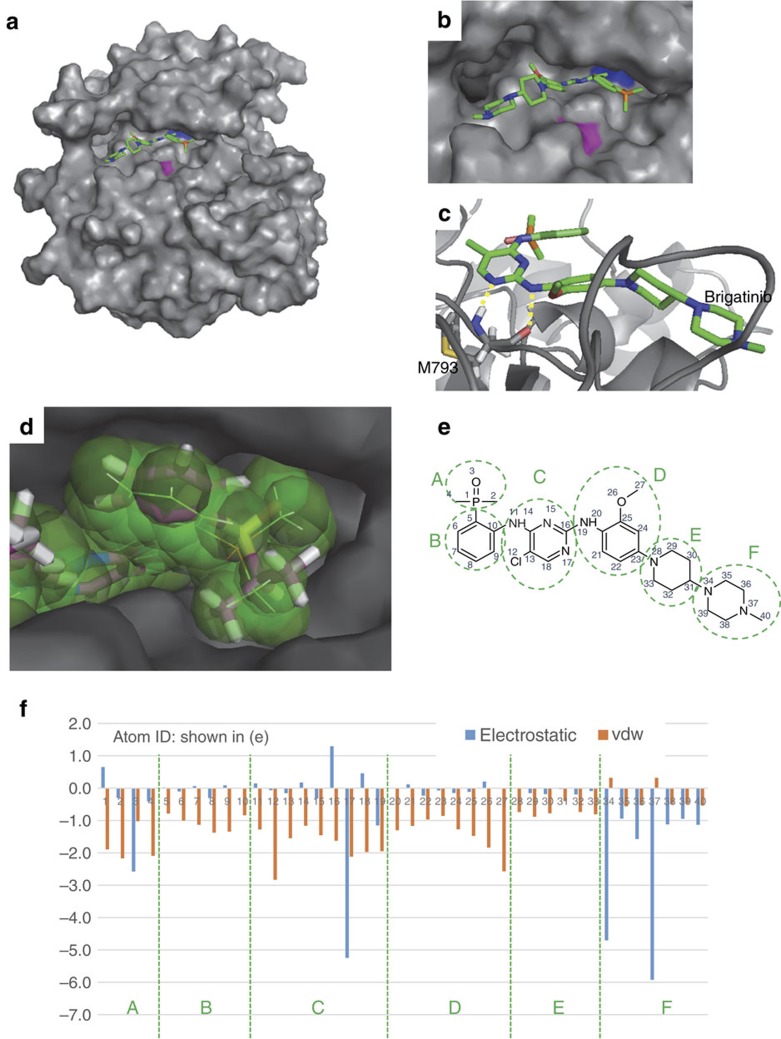
Structure model of EGFR–brigatinib interactions. (**a**,**b**) The brigatinib-binding mode for the EGFR-C797S/T790M/L858R mutant (EGFR-triple-L858R). The mean structure of the EGFR–brigatinib complex, generated by molecular dynamic simulations for 10 docking poses, is shown. EGFR was depicted by a surface model (T790M, blue; C797S, purple; others, grey), and brigatinib was depicted by sticks (C, green; N, blue; O, red; P, orange; and H, hydrogen). In the structure model, brigatinib fits into the ATP-binding pocket without a sterical crush to T790M and C797S demonstrated by overview (**a**) and zoom-in of ATP-binding pocket (**b**). (**c**) Hydrogen bonds between the triple-mutant EGFR and brigatinib. The protein backbone and M793 of EGFR-triple-L858R were depicted by a grey backbone tube and sticks (C, grey; N, blue; O, red and H, hydrogen), respectively. Hydrogen bonds were shown by dashed yellow lines. (**d**) Comparison of the inhibitor-binding mode between the EGFR–brigatinib and ALK–TAE684 complexes. TAE684 was depicted by thick sticks (C, magenta; N, blue; O, red; S, yellow; and H, hydrogen) after the crystal structure of EML4-ALK in complex with TAE684 (PDB-ID: 2XB7) was superimposed to the modelling structure of EGFR (a grey surface model) in complex with brigatinib (a space-filling model with thin sticks). (**e**) Substructure and atom IDs in the energy plot were assigned to the chemical structure of brigatinib. (**f**) The mean interaction energy between the EGFR-triple-L858R and a brigatinib atom was calculated using molecular dynamic trajectories for 10 docking poses. Negative and positive values indicate favourable and repulsive interactions, respectively.

We performed the *in silico* docking simulation and molecular dynamic simulation to assess the binding compatibility between brigatinib and the triple-L858R mutant EGFR and to clarify whether the efficacy of brigatinib against the triple-mutant EGFR depends on targeting of the ATP-binding site (Supplementary Fig. 10). In our simulations, brigatinib fitted into the ATP-binding pocket of EGFR-triple-L858R without sterically crowding T790M or C797S (Fig. 4a,b), forming two hydrogen bonds with the backbone amide of M793 (Fig. 4c). Further, the ligand-binding conformation in the docked complex model was quite similar to that in the crystal structure of EML4-ALK bound to TAE684 (PDB-ID: 2XB7). This finding is considered to be reasonable because of high similarities between both protein and chemical structures of the two inhibitors. Although these structural analyses suggest that these two inhibitors were presumed to bind to EGFR with similar orientations, activity to triple-mutant EGFR significantly differed from each other in cell growth inhibition or western blotting (Fig. 3c,e). For explanation of the difference in the inhibitory activities, the docking model hinted advantageous substructures in brigatinib for EGFR binding. In the docked EGFR–brigatinib model, the phosphine oxide group fully occupies the triphosphate-binding space in the ATP-binding site (Fig. 4d), concomitantly with meaningful gains of the electrostatic or van der Waals interaction energy for all atoms in this group (Fig. 4e,f), suggesting specific interaction with EGFR. In the case of TAE684, its substitution with the isopropylsulfonyl group might disturb the intermolecular interaction pattern and explain the superiority of brigatinib to TAE684 for triple mutation shown in this study. This interaction map between brigatinib and triple-mutant EGFR represents that the components B and E contributed less to the affinity than other compartments and were suitable for structural modification (Fig. 4f).

### Effectiveness of brigatinib in lung cancer cell lines

**Figure 5:**
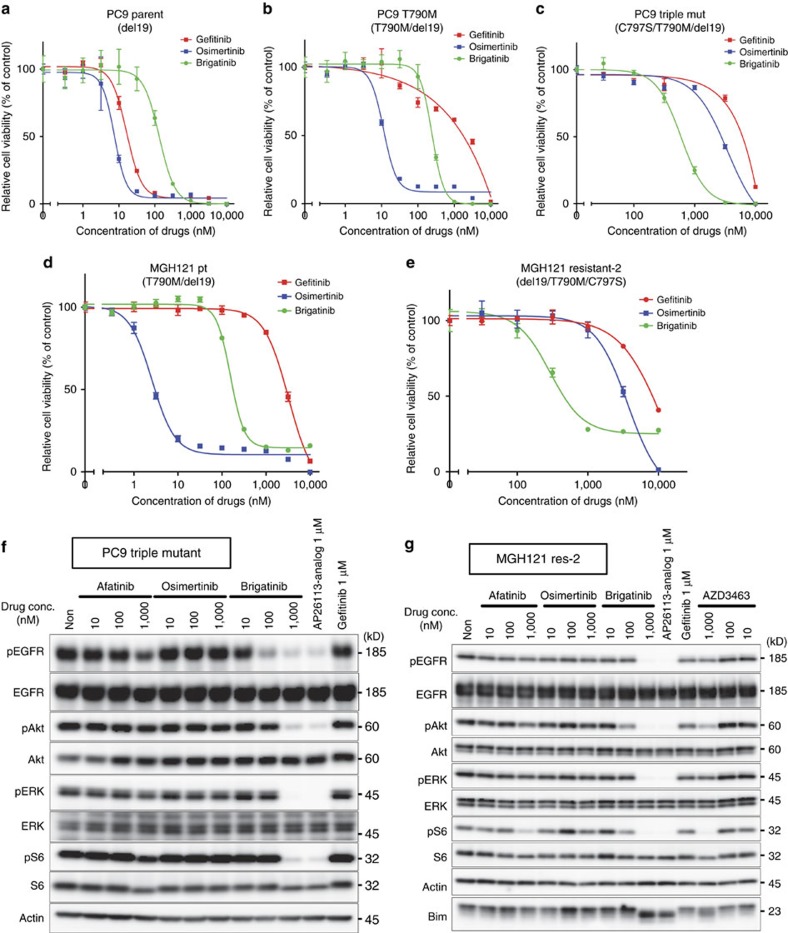
Inhibition of cell growth and downstream signal pathway in lung cancer cell lines by brigatinib. (**a**–**e**) PC9 (del19) (**a**), PC9-T790M (T790M/del19) (**b**), PC9-triple mutant (C797S/T790M/del19) (**c**), MGH121 parent (T790M/del19) (**d**) and MGH121 resistant-2 (C797S/T790M/del19) (**e**) cells were treated with serially diluted gefitinib, osimertinib and brigatinib for 72 h. Cell viability was measured using the CellTiter-Glo assay.; *N*=3. Results are expressed as mean±s.d. (**f**) Western blotting of PC9 triple mutant (C797S/T790M/del19) cells indicated that brigatinib and AP26113 analog, but not afatinib or osimertinib, suppressed phosphorylation of EGFR and its downstream signalling. (**g**) Similar results were obtained in MGH121 resistant-2.

We then evaluated the effectiveness of brigatinib and other TKIs against triple-del19-positive lung cancer cell lines. CellTiter-Glo assays demonstrated that the growth of PC9 parental cells (del19 alone) and double-mutant (T790M/del19) or triple-mutant (C797S/T790M/del19) cells was inhibited by gefitinib, osimertinib and brigatinib with a pattern similar to that observed in Ba/F3 cells expressing the corresponding mutation type (Fig. 5a–c and Table 1d). In western blotting, only brigatinib inhibited EGFR phosphorylation and its downstream signalling in PC9 triple-mutant cells. No reduction of the signal pathway in those cells was yielded by afatinib and osimertinib in contrast with observations of diminished EGFR signalling in PC9 parent and T790M cells (Fig. 5f and Supplementary Fig. 11a,b). AZD3463 showed mild activity in these PC9 cells assessed using western blotting (Supplementary Fig. 11c). Moreover, these three EGFR–TKIs showed the same pattern of effectiveness in MGH121-parental cells derived from an erlotinib-failure patient harbouring T790M/del19 and in MGH121-resistant-2 cells expressing triple-del19 established as WZ-4002–resistant MGH121 cells *in vitro*^43^ (Fig. 5d,e,g and Table 1e).

### Efficacy of brigatinib to triple-mutant EGFR *in vivo*

To confirm the superior activity of brigatinib against the triple-mutant EGFR, we performed *in vivo* experiments by administering brigatinib or osimertinib to nude mice into which EGFR-triple-del19 expressing PC9 lung cancer (PC9-triple mutant) cells had been subcutaneously injected. As a result, the mice treated with brigatinib showed significant inhibition of the growth of PC9-triple mutant cells compared with vehicle controls and osimertinib-treated group without explicit toxicity (Supplementary Fig. 12a,b). Phosphorylation of EGFR and its downstream signalling were actually inhibited by brigatinib in tumour samples obtained from mice (Supplementary Fig. 12c). This *in vivo* efficacy was confirmed in similar experiments using Ba/F3 cells expressing EGFR-triple-del19 instead of PC9 cells. Of note, osimertinib (50 mg kg^−1^) and brigatinib (75 mg kg^−1^) both successfully suppressed the growth of T790M/del19-expressing PC9 cells (Supplementary Fig. 13). These results suggested that brigatinib took advantage of triple mutation regardless of cell type and will be a promising candidate to overcome the acquired resistance of third-generation EGFR–TKIs. To attain better therapeutic effect and tumour shrinkage, it would be important to develop more potent inhibitors than brigatinib based on our structural analysis in future studies.

### The anti-EGFR antibody enhances the efficacy of brigatinib

**Figure 6:**
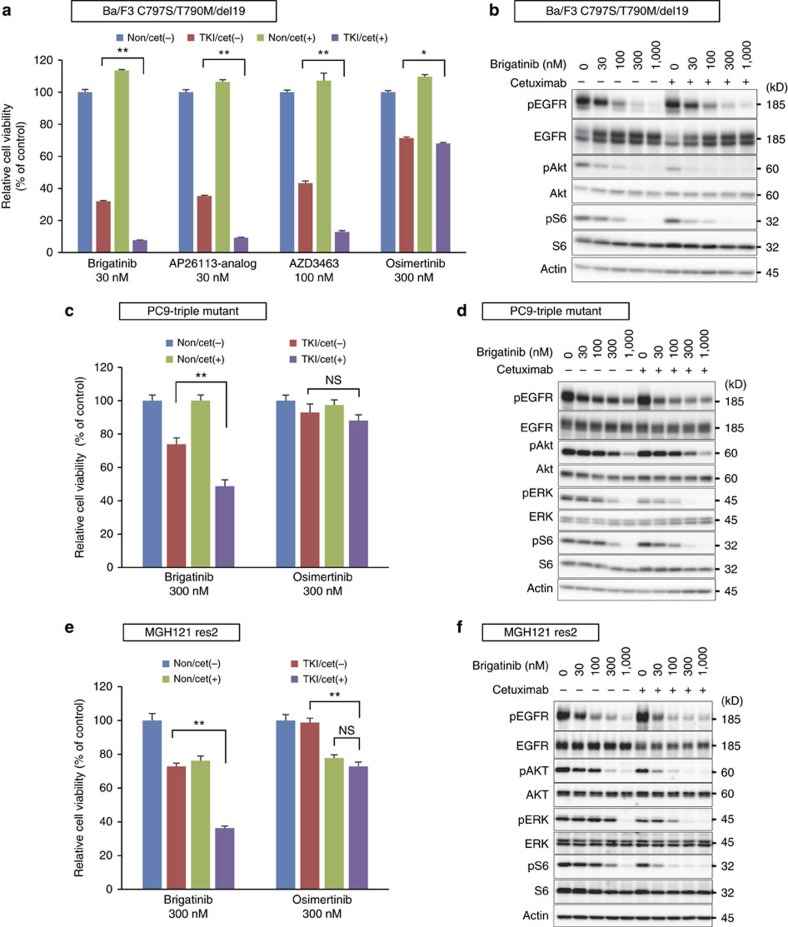
Brigatinib combined with cetuximab synergistically suppressed the growth of EGFR-C797S/T790M/del19-expressing cells *in vitro.* (**a**) The cell growth inhibition of Ba/F3 cells expressing EGFR-C797S/T790M/del19 (EGFR-triple-del19) treated with brigatinib, AP26113-analog, AZD3463 and osimertinib at indicated concentrations combined with or without cetuximab (10 μg ml^−1^) for 72 h assessed by CellTiter-Glo assay. (**b**) Inhibition of EGFR signal pathway in BaF3 EGFR-triple-del19 cells treated with brigatinib+cetuximab (10 μg ml^−1^) for 6 h was evaluated using western blotting. (**c**,**d**) The cell growth inhibition of PC9 triple-mutant cells (**c**) and MGH121-res2 cells (**d**) treated with brigatinib and osimertinib at indicated concentrations combined with or without cetuximab (10 μg ml^−1^) for 72 h assessed by CellTiter-Glo assay. (**e**,**f**) Inhibition of EGFR signal pathway in PC9 triple-mutant cells (**e**) and MGH121-res2 cells (**f**) treated with brigatinib+cetuximab (10 μg ml^−1^) for 6 h was evaluated using western blotting.; Results in **a**,**c**,**e** are expressed as mean±s.d. (*N*=3). The significance of difference between indicated groups are calculated by Student's *t*-test (NS; not significant, *P<0.05, **P<0.01).

Referring to a previous report that stated that afatinib+cetuximab combination was effective for acquired resistance of first-generation EGFR–TKI in preclinical models and patients^25^,^47^, we evaluated the combination of brigatinib and cetuximab or panitumumab against the triple-mutant EGFR. The cell viability assay using Cell-Titer Glo kit demonstrated that cetuximab enhanced the efficacy of brigatinib or AP26113-analog against triple-del19 Ba/F3 cells with approximately three-fold decrease of IC_50_, whereas no synergistic benefit was obtained in osimertinib (Fig. 6a). The potentiated inhibition of downstream pathway by combination of brigatinib with cetuximab was observed using western blotting (Fig. 6b). These effects were reproduced among triple-mutation lung cancer cell lines both in cell viability assay and western blotting analysis (Fig. 6c–f). The other anti-EGFR antibody, panitumumab in combination with brigatinib, indicated similar growth inhibition (Supplementary Fig. 14a–c).

**Figure 7:**
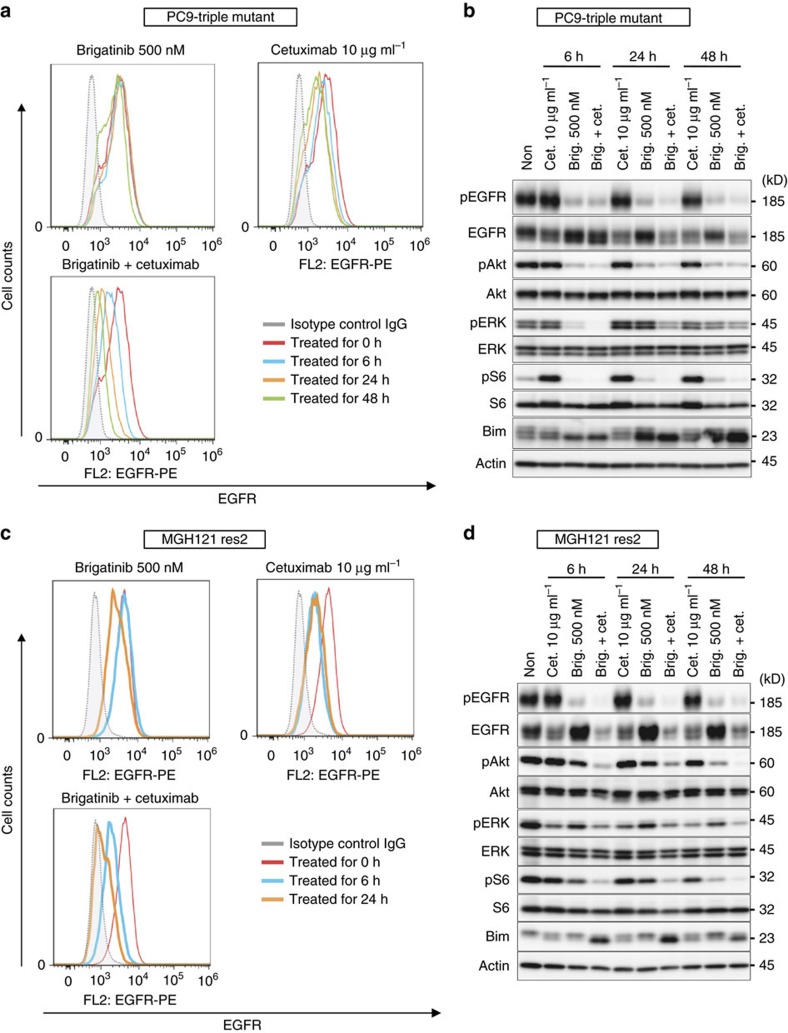
Brigatinib combined with cetuximab enhanced internalization and reduced EGFR expression. (**a**) FACS analysis using a PE-conjugated EGFR antibody of PC9 triple-mutant cells treated with brigatinib, cetuximab, brigatinib+cetuximab for 0, 6, 24 and 48 h demonstrated a time-dependent marked decrease in surface EGFR after treatment with brigatinib+cetuximab over a period of up to 48 h, and a moderate decrease with cetuximab alone. (**b**) Western blotting assessment of the cells corresponding to the treatments in **a**. (**c**,**d**) FACS analysis and western blotting performed with MGH121-res2 cells using the same method as with the PC9 triple-mutant cells in **a**,**b**.

**Figure 8:**
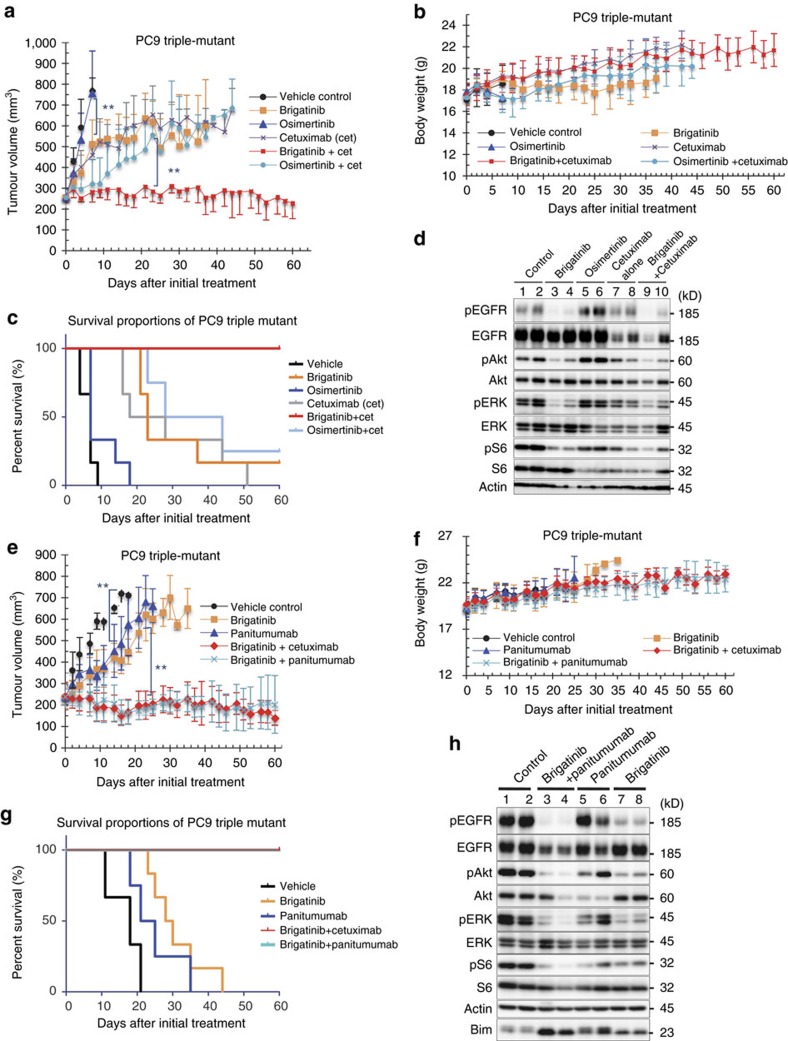
Brigatinib combined with cetuximab or panitumumab synergistically suppressed the growth of EGFR-C797S/T790M/del19-expressing cells *in vivo*. (**a**,**b**) PC9 cells expressing EGFR-C797S/T790M/del19 were subcutaneously implanted into Balb-c nu/nu mice. When the average tumour volume reached ∼200 mm^3^, the mice were randomized into vehicle control or treatment groups (50 mg kg^−1^ of osimertinib, 75 mg kg^−1^ of brigatinib, 1 mg per mouse of cetuximab three times a week or 75 mg kg^−1^ of brigatinib combined with cetuximab administered as previously described) and treated once daily by oral gavage for the indicated period. Tumour volume (*V*) was calculated as 0.5 × length × width^2^, and body weights (B.W.) of mice were measured twice weekly.; *N*=6. Results are expressed as mean±s.d. The significance of difference between the mean tumour volume of control and of brigatinib on day 7, between brigatinib and brigatinib+cetuximab on day 23, respectively, are calculated by Mann–Whitney *U* test (**P<0.01). (**c**) Survival periods of mice in each treatment arm were demonstrated using the Kaplan–Meier curve. (**d**) Phosphorylation of EGFR and its downstream signalling in two tumour samples obtained from each group were evaluated using western blotting. (**e**,**f**) *In vivo* experiment of PC9 triple-mutant cells following a similar protocol as in Fig. 8a–b, using panitumumab 0.5 mg per mouse two times a week administered peritoneally instead of cetuximab.; *N*=6. Results are expressed as mean±s.d. The significance of difference between the mean tumour volume of control and of brigatinib on day 16, between brigatinib and brigatinib+panitumumab on day 23, respectively, are calculated by Mann–Whitney *U* test (**P<0.01). (**g**) A Kaplan–Meier curve of the survival of the mice in each treatment arm. (**h**) Phosphorylation of EGFR and its downstream signalling in two tumour samples obtained from xenografts of PC9-triple mutant cells treated for 8 days with the indicated drugs (brigatinib: 75 mg kg^−1^ daily, administered orally; panitumumab: 0.5 mg per mouse two times a week, administered peritoneally) were assessed by western blotting with the indicated antibodies.

**Figure 9:**
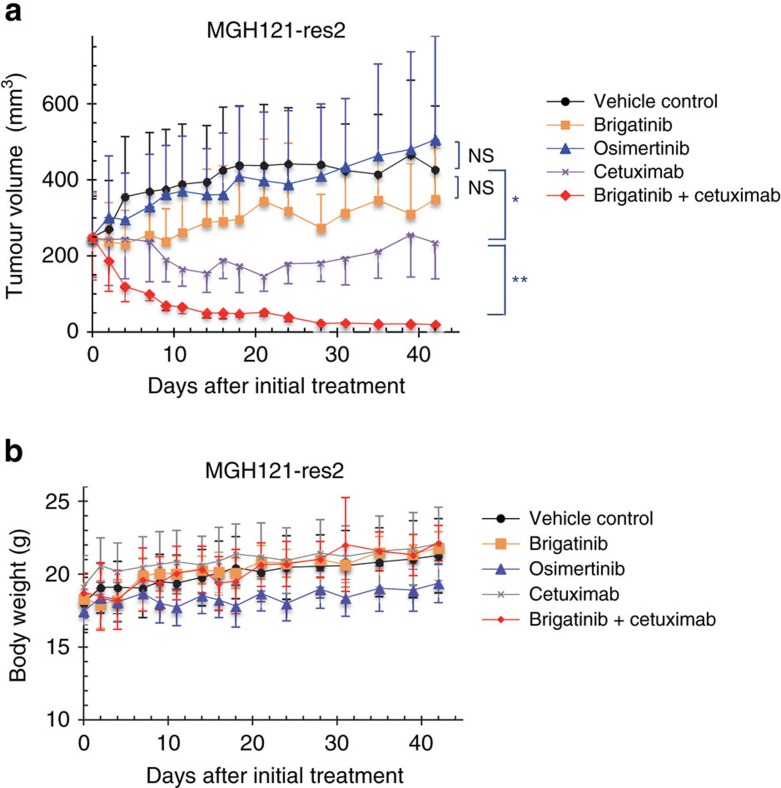
Brigatinib combined with cetuximab synergistically suppressed the growth of EGFR-C797S/T790M/del19-expressing lung cancer cells *in vivo*. (**a**,**b**) MGH121-res2 expressing EGFR-C797S/T790M/del19 were subcutaneously implanted into SCID-beige mice. When the average tumour volume reached ∼200 mm^3^, the mice were randomized into vehicle control and treatment groups (50 mg kg^−1^ of osimertinib (po), 75 mg kg^−1^ of brigatinib (po), 1 mg per mouse of cetuximab two times a week and 75 mg kg^−1^ of brigatinib combined with cetuximab administered as previously described, respectively) and treated for the indicated period. Tumour volume (*V*) was calculated as 0.5 × length × width^2^, and the body weights (B.W.) of the mice were measured twice weekly. *N*=6. Results are expressed as mean±s.d. The significance in difference between the mean tumour volume of control and of osimertinib, brigatinib and cetuximab, between cetuximab and brigatinib+cetuximab, respectively, on day 42 are calculated by Mann–Whitney *U* test (NS: not significant, *P<0.05, **P<0.01).

To further understand the benefit of combination with anti-EGFR antibody, we evaluated the cell surface expression of EGFR in PC9 triple-mutant cells after treatment with cetuximab, brigatinib and brigatinib+cetuximab in combination for 0, 6, 24 and 48 h. EGFR expression analysis by flow cytometry of the treated cells demonstrated a significant decrease over time in the cell surface EGFR level with brigatinib+cetuximab and a moderate decrease with cetuximab alone, but it demonstrated no reduction with brigatinib alone (Fig. 7a). Western blot analysis of the corresponding treated cells showed that the decrease of total cellular EGFR achieved with cetuximab was potentiated when the cells were treated with brigatinib and cetuximab in combination and that the inhibition of phosphorylation of EGFR along with downstream signalling was also enhanced by this combination (Fig. 7b). In addition, the same experiments confirmed the suppression of total and cell surface EGFR expression using triple-del19-mutated EGFR-expressing MGH121-res2 cells (Fig. 7c,d). These results suggest that synergy of brigatinib and cetuximab was induced through the degeneration of EGFR on the surface caused by cetuximab resulting in intensification of the efficacy of brigatinib. We then performed *in vivo* experiments of PC9 triple-mutant xenograft cells as described above comparing with vehicle control, brigatinib alone, osimertinib alone, cetuximab alone, combination of osimertinib and cetuximab and combination of brigatinib and cetuximab. The combination of brigatinib and cetuximab demonstrated significant suppression of tumour growth without toxicity and achieved prolongation of survival periods compared with other treatment groups, especially osimertinib-treated group without any superiority to control group (Fig. 8a–c). We confirmed that its efficacy depended on the inhibition of phosphorylation of EGFR and the decreased expression of EGFR itself in western blotting of tumour samples obtained from each treatment group (Fig. 8d). Panitumumab, another anti-EGFR antibody used for treating patients with colorectal cancer, showed a comparable synergistic effect as cetuximab in an *in vivo* experiment (Fig. 8e–g). Western blotting of resected xenografts showed that the synergy was also induced through combination with panitumumab, suggesting the importance of the concurrent anti-EGFR antibody regardless of its mode of action (Fig. 8h). Another EGFR-triple-mutated lung cancer xenograft model using MGH121-res2 cells showed not only a growth inhibition effect with brigatinib but also a significant tumour shrinkage without toxicity with a combination treatment of cetuximab and brigatinib (Fig. 9a,b).

## Discussion

In this study, we demonstrated the efficacy of brigatinib against triple-mutant EGFR-positive cells that acquired resistance even to third-generation EGFR–TKIs. Engineered Ba/F3 cells overexpressing triple-mutant EGFR were shown to be sensitive to brigatinib not only *in vitro* but also *in vivo*, as were the lung cancer cell lines with the triple-mutation *in vitro* (Figs 1 and 5). Brigatinib also demonstrated growth inhibition activity in PC9 triple-mutant xenograft model and in combination with anti-EGFR antibody to potentiate the efficacy both *in vitro* and *in vivo* as shown in first-generation EGFR–TKI-resistant patients (Figs 8 and 9 and Supplementary Fig. 12). Discovery of a promising drug that is effective against the triple mutation should be meaningful considering that the approval of osimertinib, the third-generation EGFR–TKI, in the United States and other countries may lead to a rapid increase in cases of acquired resistance due to the triple-mutant EGFR in the clinical setting.

As brigatinib is now under clinical development as an ALK–TKI^48^, we also investigated the efficacy of similarly structured ALK–TKIs against the triple-mutant EGFR. However, no other drugs exceeded brigatinib and its analog (Figs 3 and 5 and Supplementary Figs 7–9, 11). We had doubts about the disparity in their activity even if it is true that brigatinib was originally developed as a dual inhibitor of EGFR and ALK. The structure–activity relationship and computer simulation suggested that the chloro, phosphine oxide group and methoxy group of brigatinib worked as key elements that contribute to its superior efficacy for triple-mutant EGFR (Fig. 3a–c). Also, these groups meaningfully gained the electrostatic or van der Waals intermolecular interaction energy in molecular simulation (Fig. 4e,f), supporting the speculation from the structure–activity relationship. The binding pose of brigatinib also revealed that sufficient space appears to be available for substitutions on the piperidine ring and a phenyl ring connected to the phosphine oxide group, concomitantly with smaller contributions of the two substructures to the binding stability (Fig. 4e,f). These two functional groups (mentioned as parts B and E in Fig. 4e,f) may be suitable to be partially modified to achieve better binding affinity because of their lesser involvement in the predicted binding mode. These implications provide the opportunity to develop more potent new drugs by investigating derivatives of brigatinib in the future.

This study had several limitations. First, brigatinib did not demonstrate satisfactory efficacy in patients with EGFR-mutated lung cancer; a recent phase 1/2 trial reported that only two of 42 cases achieved partial response^48^. However, the plasma concentrations of brigatinib at 180 mg per day in a steady state reported previously (1,694.3 nM (ref. 48) and 1,447 nM (ref. 49)) were higher than the IC_50_ values for triple-mutant EGFR presented in our study (Figs. 1b and Table 1d,e). We expect that a combination of brigatinib and anti-EGFR antibody would improve sensitivity to the triple-mutant EGFR, resulting in better efficacy as shown in our study of the long-term tumour stability in PC9 triple-mutant xenografts and significant tumour shrinkage in MGH121-res2 xenografts; this implies that long-term ‘stable disease' or ‘partial response' was achieved with the combination therapy, whereas only the inhibition of tumour growth was attained with brigatinib monotherapy. Second, there is limited evidence that brigatinib directly affects the ATP-binding site of the triple-mutant EGFR because of the absence of a co-crystal structure, although our *in vitro* kinase assay results suggest that brigatinib inhibits triple-mutant EGFR in an ATP-competitive manner, and our computer simulation demonstrated its compatible interaction with the triple-mutant EGFR. Third, we have not yet experienced a sufficient number of cases of osimertinib resistance to estimate the true number of the triple-mutant EGFR. Up to the present, the prevalence of the triple mutation was estimated to be 2–4% among patients with lung cancer according to the frequency of 22% among osimertinib-resistant cases reported in WCLC2015 (ref. 50), and the C797S population would be equal to that of ALK-rearranged lung cancer patients with a similar clinical magnitude. In addition, in our N-ethyl-N-nitrosurea mutagenesis drug screen, all clones resistant to osimertinib mediated only the C797S mutation (unpublished data). Unless the triple-mutant EGFR occurs less frequently than is currently expected (∼20%), beating the triple-mutant EGFR is a worthy challenge as it is highly refractory to available drugs, although we can hope to overcome resistance due to other mechanisms such as T790M loss or bypass pathway activation using existing treatment modalities, for example, turning back to first-generation EGFR–TKIs or combination therapy.

In conclusion, we found brigatinib to be potent against the triple-mutant EGFR in a focused drug-screening protocol and confirmed its activity in *in vivo* and *in vitro* assays. The activity depended on ATP-competitive manner with less affection to wild-type EGFR. The combination therapy with anti-EGFR antibody showed more preferable activity than monotherapy *in vitro* and *in vivo* without toxicity. The structure–activity relationship demonstrated that the efficacy of brigatinib exceeded that of its analogs, identifying several key basic structure and functional groups, such as the phosphine oxide group predicted from the computer simulation. The simulation indicated not only the conformation of brigatinib binding to the triple-mutant EGFR ATP-binding pocket in a compatible manner but also the potential for developing a more potent inhibitor by partial substitution in future direction. While the potency of brigatinib monotherapy does not seem to be completely satisfactory, the combination with anti-EGFR antibody strikingly reduced IC_50_ against triple-mutant EGFR by inducing the degradation of surface and total EGFR expression, leading to tumour shrinkage of the triple-mutant EGFR-harbouring cells and significant prolongation of the survival of xenograft-bearing mice. Given that the development of brigatinib is now in the late phase of clinical trials and anti-EGFR antibodies are now broadly used clinically for several cancers other than lung cancer, the findings of this study should help to overcome the acquired resistance to third-generation EGFR–TKIs.

## Methods

### Kinase inhibitors and other drugs

The drugs used in the experiments and the companies from which they were purchased are shown in Supplementary Table 1.

### Reagents and cell culture

MGH121 (EGFR-T790M/del19 derived from a lung cancer patient), MGH121-resistant-2 (EGFR-C797S/T790M/del19), PC9 parent (amplified EGFR-del19), PC9 T790M (amplified EGFR-T790M/del19) and PC9 triple-mutant (amplified EGFR-C797S/T790M/del19) cells were cultured in RPMI with 10% serum. These cells were kindly provided by Drs. Niederst and Engelman. Ba/F3 cells harbouring EGFR mutations were cultured in low-glucose Dulbecco's minimal essential medium (DMEM) with 10% fetal bovine serum (FBS). The MGH121 resistant-2 cells were established from MGH121 by treating with 1 μM WZ-4002, and the PC9 T790M cells were obtained from PC9 parental cells by treating with 1 μM gefitinib as *de novo* persistent resistant clone^43^. The PC9 triple mutant cells were generated by lentivirus infection of EGFR-triple-del19 from the PC9 parental cells. All cells were routinely tested and verified to be free of mycoplasma contamination.

### Generating lentivirus and stable expression in Ba/F3 cells

The full-length wild-type EGFR was synthesized from cDNA obtained from A549 cells. The EGFR-activating mutation, del19 or L858R, was obtained from the cDNA of EGFR-exon 19 deletion (del19)-harbouring HCC827 cells or L858R-harbouring lung cancer specimens, respectively. Each EGFR was amplified using polymerase chain reaction and then cloned into a pENTR vector. The T790M and/or C797S mutants were generated by QuikChange site-directed mutagenesis using the following primers: T790M F- 5′-CCGTGCAGCTCATCATCCAGCTCATGCCCTTC-3′, and T790M R- 5′-GAAGGGCATGAGCTGCATGATGAGCTGCACGG-3′, C797S F- 5′-CATGCCCTTCGGCTCCCTCCTGGAGCTA-3′, and C797S R – 5′-TAGTCCAGGAGGGAGCCGAAGGGCATG-3′.

The resulting pENTR-EGFR mutation constructs were sequenced and used as a template to make the pLenti6.3 lentiviral vector using LR clonase II. The lentivirus was made by transfecting the pLenti6.3 constructs along with helper plasmids (ViraPower) in 293FT cells. Virus production, collection and infection were completed following the manufacturer's protocol. The Ba/F3 cells were selected by culturing for 1 week with 7 μM blasticidin in DMEM with interleukin-3 (IL-3)-supplemented 10% FBS and then in DMEM without IL-3 to obtain the EGFR signalling-addicted cells.

### Cell viability assays

Three-day cell viability assays were carried out by plating 2,000, 1,500 and 2,000 cells per well of Ba/F3, PC9 or MGH121, respectively, into black transparent-bottom 96-well plates. On the same day for Ba/F3 cells and the following day for PC9 and MGH121 cells, the cells were treated with each TKI across a 10-dose range from 0.3 nmol l^−1^ to 10 μmol l^−1^. After 72 h of drug treatment, cell viability was measured using the CellTiter-Glo assay (Promega).

### Drug sensitivity screening

Ba/F3 cells of EGFR-del19, -T790M/del19, -C797S/del19 and -C797S/T790M/del19 were plated with 2,000 cells per well into black 96-well plates and treated for 3 days with a panel of 30 inhibitors including dimethylsulfoxide (DMSO) controls prepared in-house. After the incubation, cell viability was measured using CellTiter-Glo assay. The relative cell viability was calculated as a ratio of each value to that of the DMSO control. Experiments were repeated three times independently, and the average relative cell viability was calculated and shown as a heat map (Original data are available in Supplementary Data 1).

### Antibodies and western blotting

Tumour tissues or cells grown under the specified conditions were washed with cold PBS before addition of the SDS lysis buffer (100 mM Tris, 1% SDS, 10% glycerol). Lysates were transferred to microtubes, boiled for 5 min at 100 °C and then vortexed. Protein quantification was performed using the BCA Protein Assay Reagent (Pierce) according to the manufacturer's protocol. Western blot analyses were conducted after separation by SDS-PAGE and transferred to polyvinylidene difluoride membranes. After blocking in 5% BSA with Tris-buffered saline/Tween 20 (TBS-T) or 5% skim milk/TBS-T, membranes were incubated with phospho-EGFR antibody (Tyr1068; Abcam, ab5644, 1:1,000), total EGFR (Cell Signaling Technology, #4267, 1:2,000), phospho-Akt (Ser473; Cell Signaling Technology, #4060, 1:1,000), total Akt (Cell Signaling Technology, #4691, 1:5,000), phospho-ERK (Thr202/Tyr204; Cell Signaling Technology, #9101, 1:5,000), total ERK1/2 (Cell Signaling Technology, #9102, 1:2,000), phospoh-S6 (Ser240/244, Cell Signaling Technology, #5364, 1:10,000), total S6 (Cell Signaling Technology, #2217, 1:2,000) or β-actin (Sigma-Aldrich, A5228, 1:10,000).

### *In vitro* kinase assay of EGFR protein and inhibitors

The recombinant proteins of the kinase domain of wild-type EGFR and EGFR-C797S/T790M/L858R were purchased from Signal Chem. The inhibitors were purchased as described earlier. The appropriate amount of target proteins calculated on the basis of the ADP-Glo assay manufacturer's protocol was incubated in 96-well half area white plates with serially diluted inhibitor over a 10-dose range from 0.0002, nM to 10 μM for 10 min at room temperature. ATP at concentration of 1, 10, 100 and 1,000 nM was mixed with 100 μg ml^−1^ substrate and added to a kinase protein–inhibitor mixture, and then incubated for 60 min at room temperature. After the kinase reaction, an equal volume of ADP-Glo Reagent was added to terminate the kinase reaction, and the remaining ATP was depleted. The Kinase Detection Reagent was added both to convert ADP to ATP and to allow the newly synthesized ATP to be measured using the luciferase/luciferin reaction. The light generated was measured using a luminometer.

### Flow cytometry

To evaluate the level of EGFR, fluorescence-activated cell sorting (FACS) analysis was performed with PE Mouse Anti-Human EGF receptor (BD Biosciences, #555997, 100 μl per 1 × 10^6^ cells) on Cytomics FC500 (BECKMAN COULTER). The assessed cells were incubated with cetuximab 5 ng for 5 min before the addition of the PE antibody to cancel the cross-activity with cetuximab.

### *In vivo* evaluation of brigatinib and osimertinib

All mouse studies were conducted through Institutional Animal Care and Use Committee-approved animal protocols according to the institutional guidelines. PC9-EGFR- C797S/T790M/del19 (PC9-triple-mutant) cells (6 × 10^6^) or PC9-EGFR-T790M/del19 (PC9-T790M) cells (6 × 10^6^) were suspended in 100 μl of 1:2 Matrigel and subcutaneously implanted into Balb-c *nu/nu* mice (Charles River). Tumour growth was monitored twice weekly by bilateral caliper measurement, and tumour volume was calculated as 0.5 × length × width × width (mm^3^). When the average tumour volume reached ∼200 mm^3^, the mice were randomized into vehicle and treatment groups using the restricted randomization such that the mean tumour size of each group was equivalent (control, 50 mg kg^−1^ of osimertinib, or 75 mg kg^−1^ of brigatinib, respectively). The mice whose implanted tumour size was ranked in the top 5% and bottom 5% were excluded from randomization to minimize the variation of tumour sizes. The mice were treated once daily by oral gavage for the indicated period. Relative tumour volume was calculated by dividing by the tumour volume on day 0. The body weights of the mice were measured twice weekly. Implanted tumours were resected from the mice on day 15 of drug treatment and were fixed with formalin. The mice were euthanized when the tumour size exceeded 700 mm^3^ within several days. The investigators performing tumour measurements were not blinded to treatment groups. The sample size (minimum n=6 per treatment group) was selected to ensure satisfactory inter-animal reproducibility. The Mann–Whitney *U* test was used for the statistical analysis of the mice experiments.

### Molecular docking simulation

The genetic algorithm-docking programme GOLD 5.2 was used to perform the molecular docking of brigatinib towards the C797S/T790M/L858R triple-mutant EGFR. The standard default settings for the genetic algorithm were used. A protein structure for the docking simulation was set to the crystal structure of T790M-mutant EGFR in complex with WZ4002 (PDBID: 3IKA), which shares the largest common basic structure with brigatinib. The structure of a disordered loop (residues Leu989 – Asp1003) and the side chains of Ser797 and Arg858 were modelled using the Structure Preparation module in Molecular Operating Environment (MOE, Chemical Computing Group, Montreal, Canada) version 2013.08 (ref. 51). A compound-binding site in the triple-mutant EGFR was defined to include all atoms within 10 Å of the midpoint of Leu718 Cγ and Gly796 Cα atoms. Brigatinib was docked into the ATP-binding site with positional restraint on the common basic structure, assuming that this substructure has a similar binding geometry between brigatinib and WZ4002.

### Molecular dynamics simulation of the C797S/T790M/L858R

Ten kinds of representative binding poses of brigatinib into the C797S/T790M/L858R triple-mutant EGFR were extracted by the docking simulation and used as initial structures of the molecular dynamics (MD) simulation. Brigatinib conformation was optimized, and the electrostatic potential was calculated at the HF/6-31G* level using the GAMESS programme^52^, after which the atomic partial charges were obtained by the RESP approach^53^. The other parameters for the compound were determined by the general Amber force field^54^ using the antechamber module of AMBER Tools 12. The Amber ff99SB-ILDN force field was used for protein and ions^55^ and TIP3P was used for water molecules^56^. Water molecules were placed around the complex model with an encompassing distance of 8 Å to form a 83 × 78 × 68 Å^3^ periodic box, including roughly 13,000 water molecules. Charge-neutralizing ions were added to neutralize the system. All MD simulations were carried out using the GROMACS 4 programme^57^ on High-Performance Computing Infrastructure (HPCI). Electrostatic interactions were calculated using the particle mesh Ewald method^58^ with a cutoff radius of 10 Å. Van der Waals interactions were cutoff at 10 Å. The P-LINCS algorithm was employed to constrain all bond lengths^59^. After the fully solvated system was energy-minimized, the system was equilibrated for 100 ps under constant volume and run for 100 ps under constant pressure and temperature, with positional restraints on protein heavy atoms and compound atoms. Each production run was conducted for 50 ns under constant pressure and temperature condition without the positional restraints. In this procedure, the temperature was maintained at 298 K using velocity rescaling with a stochastic term^60^ and the pressure was maintained at 1 bar with the Parrinello–Rahman pressure coupling^61^, where the time constants for the temperature and pressure couplings to the bath were 0.3 and 1 ps, respectively. Ten independent MD simulations were performed with the different initial structures, and thus we carried out MD simulations of 500 ns in total. All MD runs were carried out with time steps of 2 fs and snapshots were output every 2 ps to yield 500 snapshots per nanosecond of simulation.

### Data and statistical analysis

Data were analysed using GraphPad Prism software (GraphPad Software). In cell growth inhibition experiments analysis, the curves were fitted using a nonlinear regression model with a sigmoidal dose response. Unless otherwise specified, data displayed are mean±s.d. Pairwise comparisons between groups (for example, experimental versus control) were made using paired or unpaired Student's *t*-tests as appropriate. Significant probability (*P*)-values are indicated as ***P<0.001, **P<0.01 and *P<0.05.

### Data availability

The authors declare that all the other data supporting the findings of this study are available within the article and its Supplementary Information files (The original data of Fig. 1a is presented in the Supplementary Data 1. The uncropped scans of the most important blots are shown in Supplementary Figs 15–21).

## Additional information

**How to cite this article:** Uchibori, K *et al*. Brigatinib combined with anti-EGFR antibody overcomes osimertinib resistance in EGFR-mutated non-small-cell lung cancer. *Nat. Commun.* **8**, 14768 doi: 10.1038/ncomms14768 (2017).

**Publisher's note:** Springer Nature remains neutral with regard to jurisdictional claims in published maps and institutional affiliations.

## Supplementary Material

Supplementary Information Supplementary Figures and Supplementary Table

Supplementary Data 1 Original dataset of Figure 1a.

Peer Review File

## Figures and Tables

**Table 1:** IC_50_ values (nM) for the mutant EGFR-expressing Ba/F3 cells, PC9 cells or MGH121 cells.

**(**a**) IC_50_ values of Ba/F3 cells expressing EGFR-del19 series to EGFR–TKIs**					
**Ba/F3-EGFR-**	**Gefitinib**	**Afatinib**	**Osimertinib**	**EGF-816**	
Del19	5.9	<0.3	1.7	2.9	
T790M/del19	5,603	78.2	6.7	18.5	
C797S/del19	2.7	2.1	513.4	1,241	
C797S/T790M/del19	2,922	392.7	740.5	1,408	
Parent(+IL-3)	>10,000	381.3	752.4	–	

**(**b**) IC_50_ values of Ba/F3 cells expressing EGFR-L858R series to EGFR–TKIs**					
**Ba/F3-EGFR-**	**Gefitinib**	**Afatinib**	**Osimertinib**	**EGF-816**	
L858R	10.4	<0.3	3	7.7	
T790M/L858R	5,922	39.2	5.8	15.5	
C797S/L858R	15.5	7.7	1,115	1,659	
C797S/T790M/L858R	>10,000	804.2	1,171	2,278	

**(**c**) IC_50_ values of PC9 cells to EGFR–TKIs**					
	**Gefitinib**	**Afatinib**	**Osimertinib**		
PC9 parent	16.38	0.638	7.463		
PC9 T790M	14,684	61.94	11.31		
PC9 triple	>10,000	1,130	3,461		

**(**d**) IC_50_ values of PC9 cells to ALK–TKIs**				
	**Brigatinib**	**AP26113-analog**	**AZD3463**	
PC9 parent	132.8	46.18	253	
PC9 T790M	243.8	89.28	378.3	
PC9 triple	599.2	194.7	622.4	

**(**e**) IC_50_ values of MGH121 cells to EGFR–TKIs**				
	**Gefitinib**	**Osimertinib**	**Brigatinib**	
MGH121-pt	3,228	2.58	155.3	
MGH121-res2	>10,000	7,155	592.1	

EGFR, epidermal growth factor receptor; TKIs, tyrosine kinase inhibitors;

(**a**–**b**) IC_50_ values for the Ba/F3 cells expressing EGFR-activating mutations with or without resistant mutations, the del19 series (**a**) and the L858R series (**b**), respectively, treated with the indicated EGFR–TKIs. (**c**–**d**) IC_50_ values for PC9 cells (parental, T790M or C797S/T790M/del19-induced) treated with the indicated EGFR–TKIs (**c**) or ALK–TKIs (**d**). (**e**) IC_50_ values for the MGH121 parental and res-2 cells treated with the indicated TKIs.
