# Supplementary material for: Brigatinib combined with anti-EGFR antibody overcomes osimertinib resistance in EGFR-mutated non-small-cell lung cancer
Source: Nat Commun. 2017 Mar 13;8:14768. doi: 10.1038/ncomms14768 (PMC5355811; doi:10.1038/ncomms14768)
Supplement: Supplementary Information — Supplementary Figures and Supplementary Table [file ncomms14768-s1.pdf]

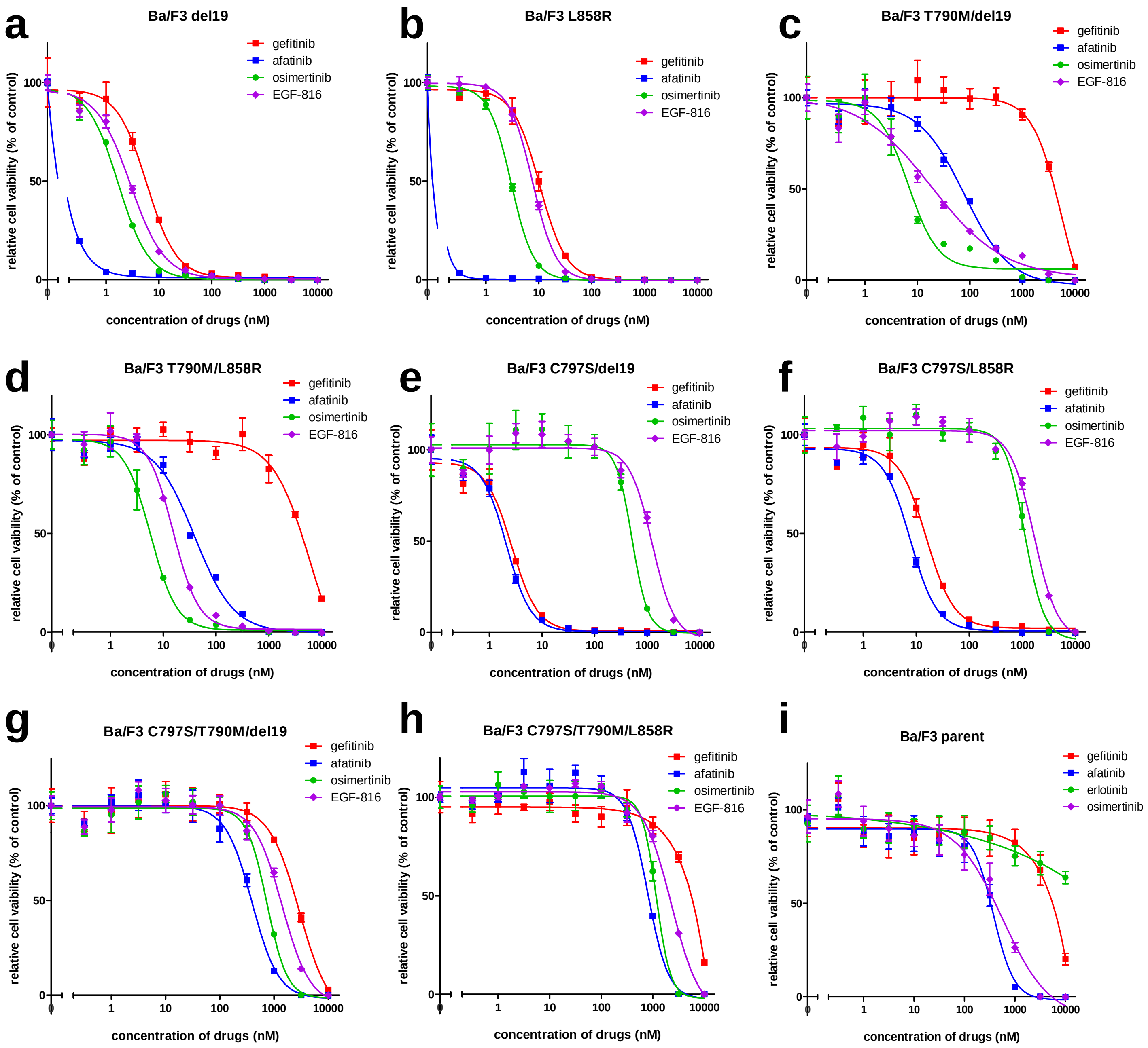

Supplementary Figure 1:**Ba/F3 cells expressing the EGFR activating-mutation alone and combined with T790M, C797S and C797S/T790M, treated with clinically relevant epidermal growth factor receptor tyrosine kinase inhibitors (EGFR–TKIs)**
(**a–i**) Ba/F3 cells stably expressing the EGFR activating-mutation alone [del19 (**a**), L858R (**b**)], the T790M/activating-mutation [del19 (**c**), L858R (**d**)], the C797S/activating-mutation [del19 (**e**), L858R (**f**)], C797S/T790M/activating-mutation [del19 (**g**), L858R (**h**)], and parent (**i**) were treated with the indicated concentrations of EGFR–TKIs for 72 h. The CellTiter-Glo assay was used to measure cell viability. ; *N*=3. Results were expressed as mean ± s.d.

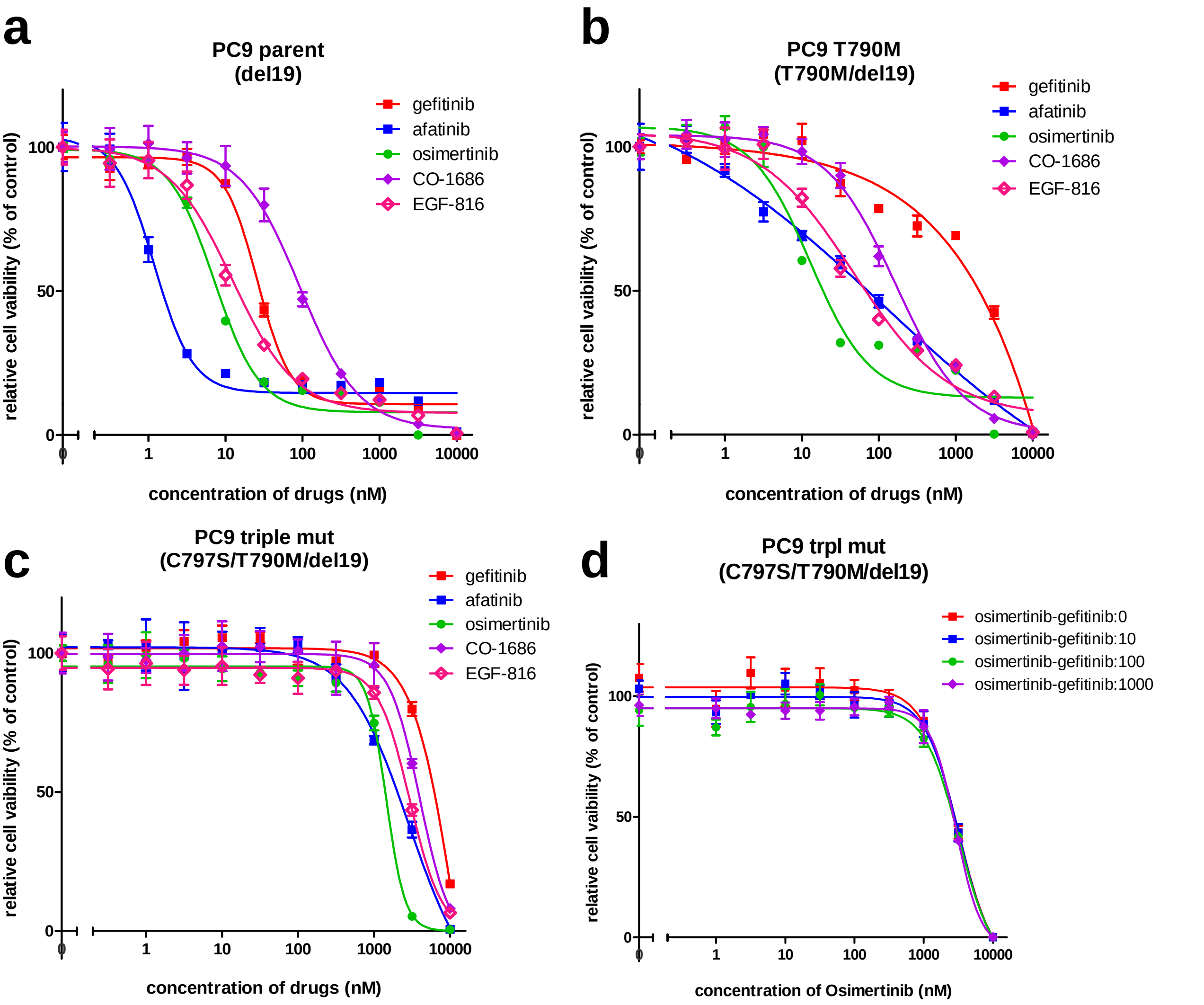

Supplementary Figure 2: Cell growth inhibition of PC9 parent, T790M, triple-mutant cells by epidermal growth factor receptor tyrosine kinase inhibitors (EGFR–TKIs). PC9 triple mutant treated with combination of gefitinib and osimertinib.

(**a–c**) PC9 parental (expressing del19) (**a**), PC9 T790M (expressing T790M/del19) (**b**), PC9 triple mutant (expressing C797S/T790M/del19) (**c**) cells were treated with the indicated concentrations of first-, second- and third-generation EGFR–TKIs for 72 h. The CellTiter-Glo assay was used to measure cell viability. (**d**) PC9 triple-mutant cells were treated with a combination of osimertinib and gefitinib. The concentrations of the concomitant gefitinib were 0, 10, 100, and 1000 nM. ; *N*=3. Results were expressed as mean ± s.d

a

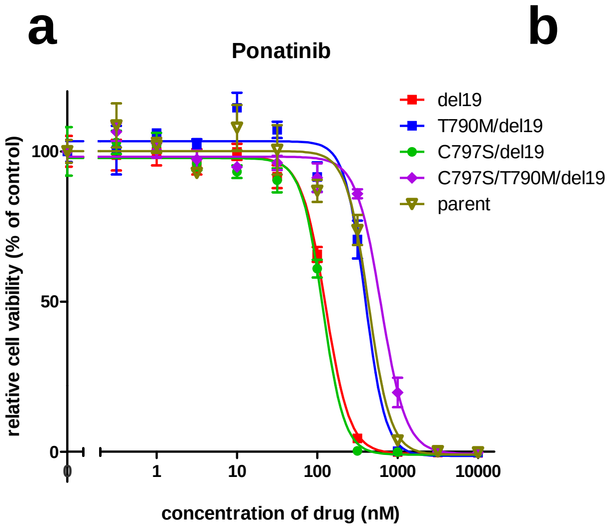

b

Ponatinib	IC_{50} (nM)
del19	126.5
T790M/del19	393.8
C797S/del19	116.3
C797S/T790M /del19	620.9
parent	431.9

c

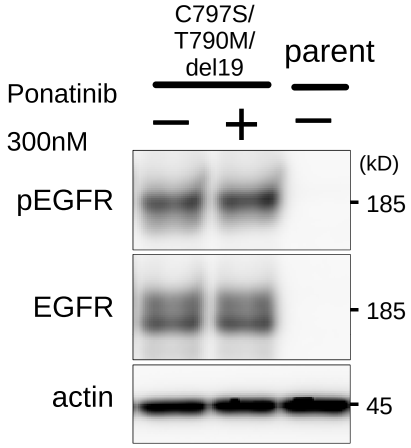

Supplementary Figure 3: Ponatinib against EGFR-triple-mutation.
(**a**) Ba/F3 cells expressing four mutation types of EGFR-del19 were treated with the indicated concentrations of ponatinib for 72 h. The CellTiter-Glo assay was used to measure cell viability. ; *N*=3. Results were expressed as mean ± s.d. (**b**) IC_{50} values were obtained from same experiment. (**c**) Western blotting of Ba/F3 cells expressing the EGFR-C797S/T790M/del19 and parent indicated that treatment with 300nM of ponatinib for 6 h showed no inhibitory activity of phosphorylation of EGFR.

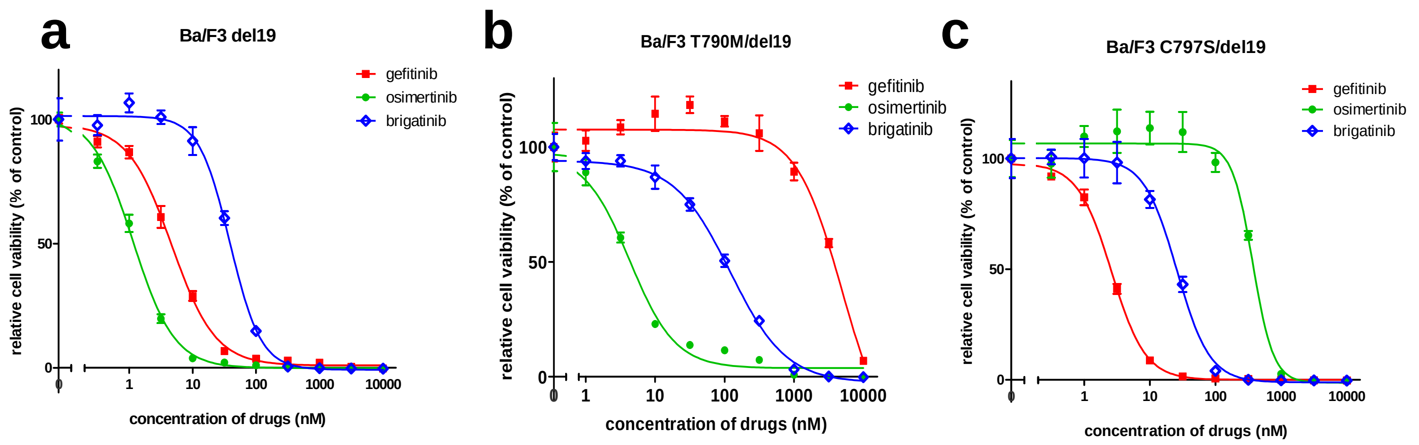

d

IC50 (nM)	Gefitinib	Osimertinib	Brigatinib
del19	5.9	1.7	39.4
T790M/del19	4845	4.3	120.2
C797S/del19	2.7	513.4	38.2

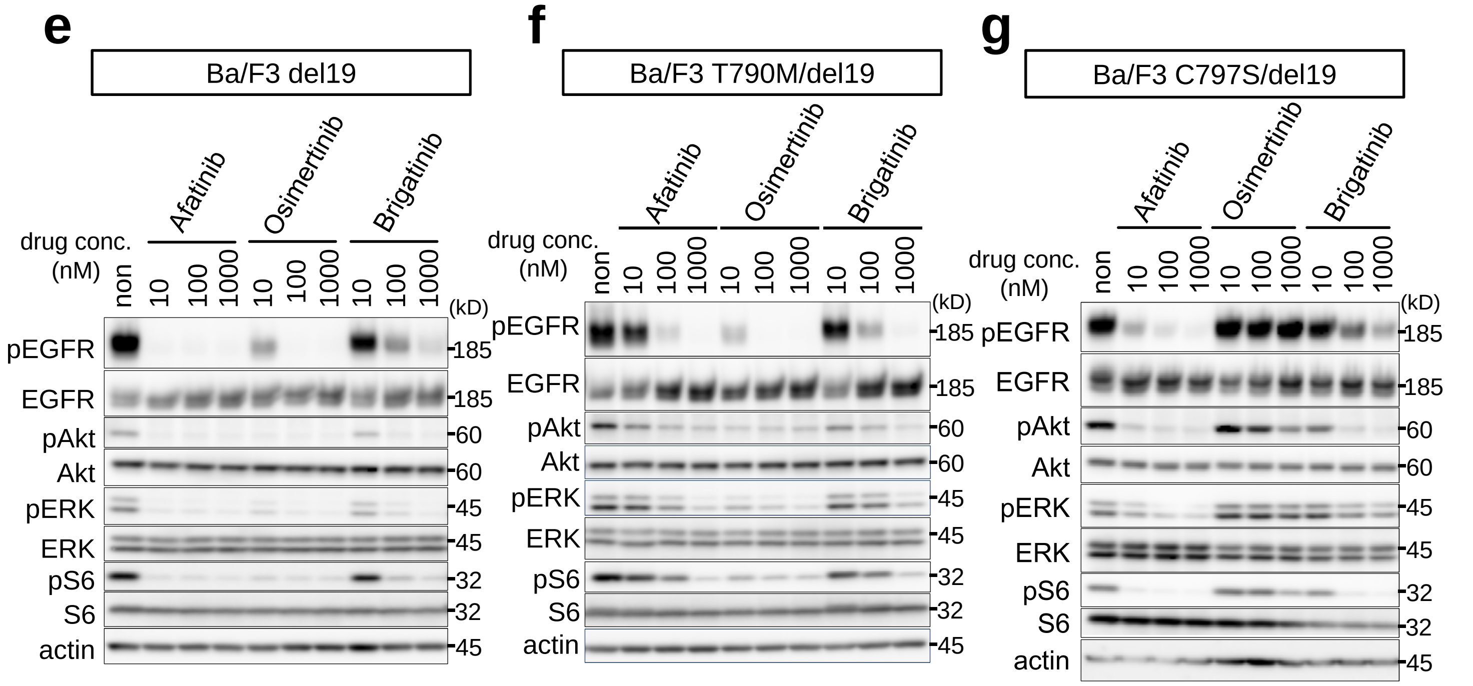

Supplementary Figure 4: Inhibition of cell growth and signal pathway in del19, T790M/del19 and C797S/del19 mutated Ba/F3 cells.
(**a**–**c**) Ba/F3 cells stably expressing the EGFR-del19 alone (**a**), T790M/del19 (**b**) and C797S/del19 (**c**) were treated with the indicated concentrations of gefitinib, osimertinib, and brigatinib for 72 h. The CellTiter-Glo assay was used to measure cell viability. ; *N*=3. Results were expressed as mean ± s.d. (**d**) IC_{50} values were calculated using those results. (**e**–**g**) Phosphorylation of EGFR and its downstream signals in Ba/F3 cells expressing EGFR-del19 alone (**e**), T790M/del19 (**f**) and C797S/del19 (**g**) treated by afatinib, osimertinib, and brigatinib for 6 h were evaluated using western blotting with the indicated antibodies.

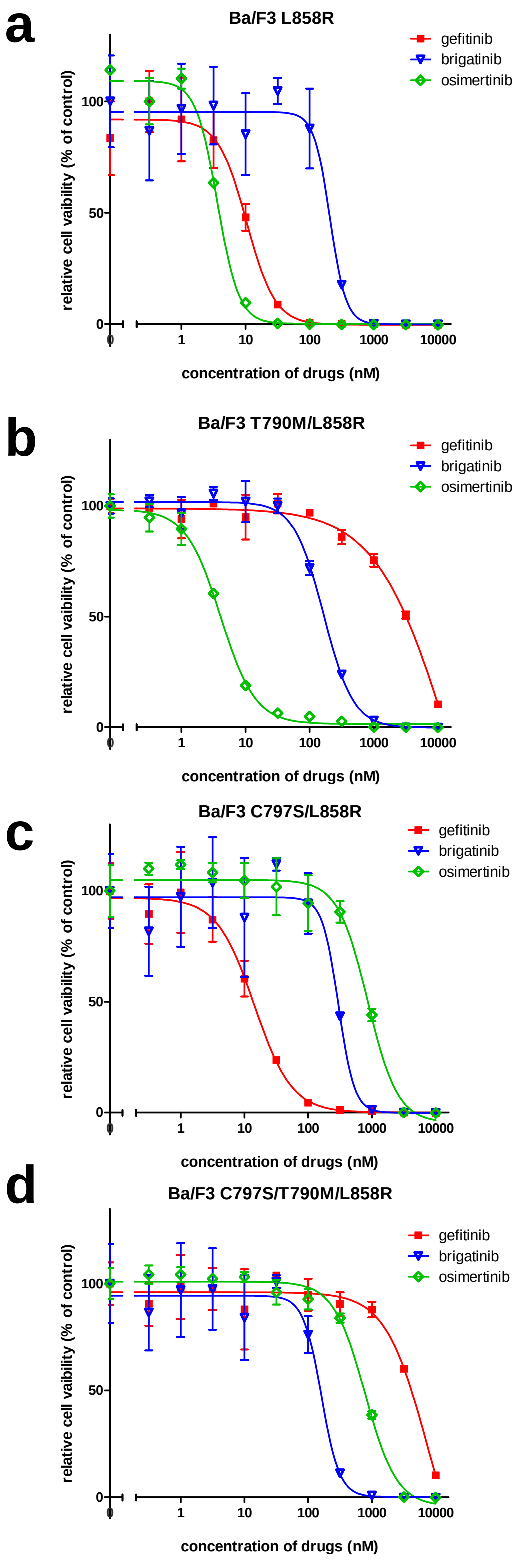

e

IC_{50} (nM)	Gefitinib	Osimertinib	Brigatinib
L858R	10.4	3.6	207.0
T790M/L858R	>10000	4.1	165.1
C797S/L858R	14.3	858.0	297.6
C797S/T790M /L858R	7176	782.4	161.5

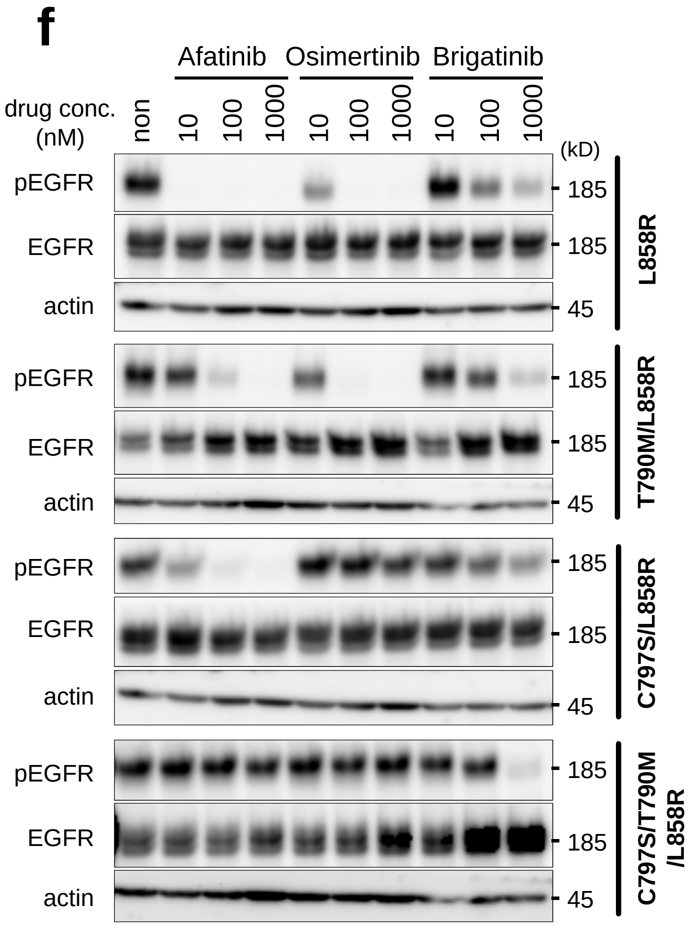

Supplementary Figure 5: Inhibition of cell growth and downstream signaling in Ba/F3 cells expressing EGFR activated by L858R treated with EGFR–TKIs and brigatinib

(**a**-**d**) Ba/F3 cells stably expressing EGFR-L858R (**a**), -T790M/L858R (**b**), -C797S/L858R (**c**) or -C797S/T790M/L858R (**d**) were treated with indicated concentrations of gefitinib, osimertinib, and brigatinib for 72h. Cell viability was assessed by CellTiter-Glo. ; *N*=3. Results were expressed as mean ± s.d. (**e**) IC_{50} values were calculated using those results. (**f**) Phosphorylation of EGFR in each mutation type of L858R introduced Ba/F3 cells treated with afatinib, osimertinib or brigatinib was assessed using western blotting.

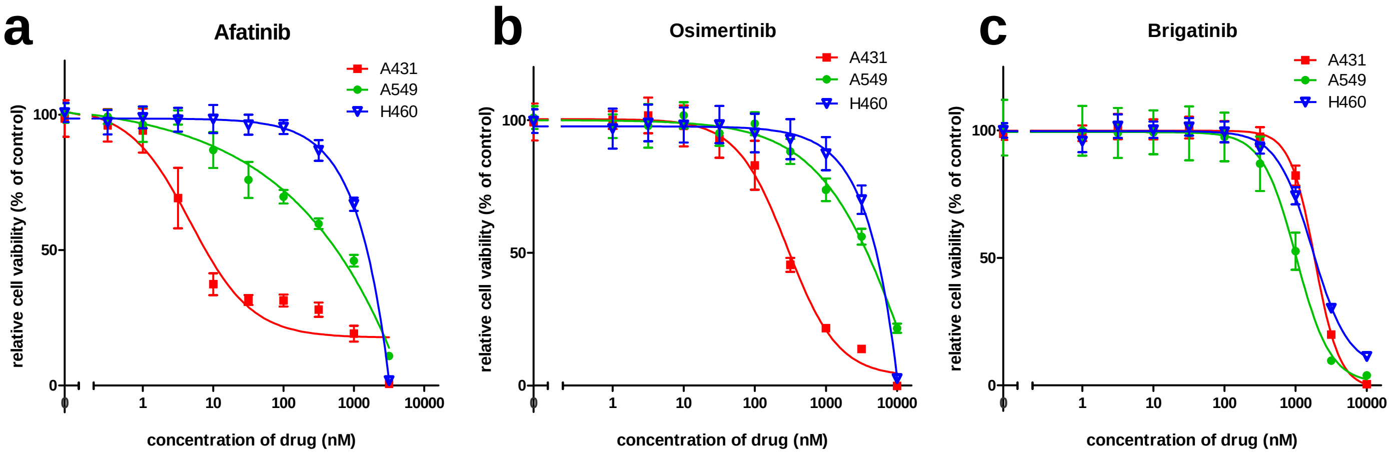

Supplementary Figure 6: Inhibition of cell growth in non-EGFR mutated cells treated with EGFR–TKIs and brigatinib

(**a–c**) A431 (EGFR amplification), A549 (KRAS mutation) or H460 (KRAS mutation) cells were treated with the indicated concentration of afatinib (**a**), osimertinib (**b**), and brigatinib (**c**) for 72h. Cell viability was assessed by CellTiter-Glo assay. ; *N*=3. Results were expressed as mean ± s.d.

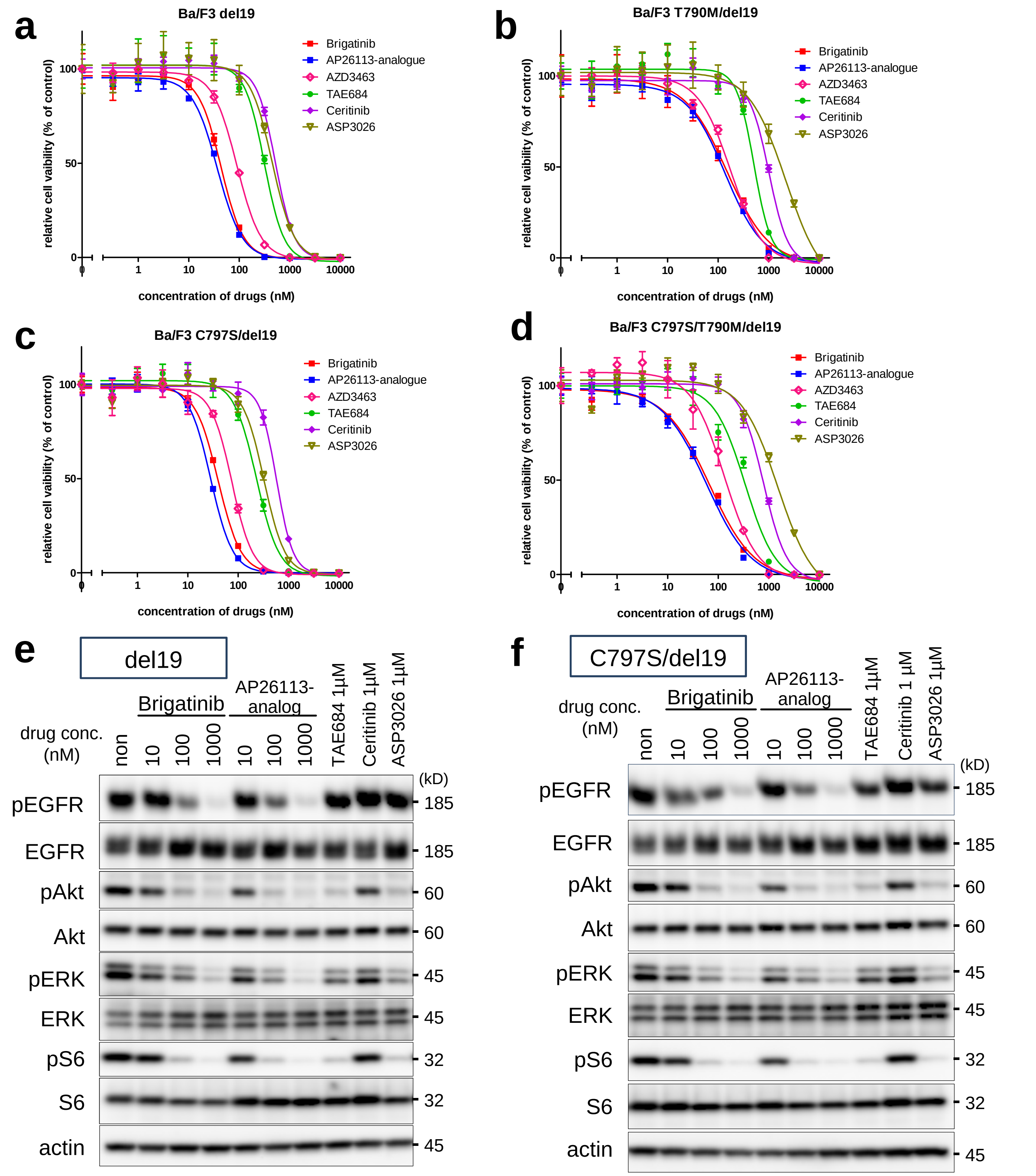

Supplementary Figure 7: Efficacy of brigatinib and similarly structured ALK–TKIs to Ba/F3 cells expressing mutant EGFR activated by del19.

(**a**–**d**) Cell growth inhibition assessed using the CellTiter-Glo assay of EGFR-mutated Ba/F3 cells [del19 alone (**a**), T790M/del19 (**b**), C797S/del19 (**c**), C797S/T790M/del19 (**d**)] treated with brigatinib, AP26113-analog, AZD3463, TAE684, ceritinib, and ASP3026 for 72 h. ; *N*=3. Results were expressed as mean ± s.d. (**e**–**f**) Western blotting of Ba/F3 cells expressing EGFR-del19 (E) and C797S/del19 (F) treated with the indicated drugs for 6 h showed that brigatinib and AP26113-analog inhibited phosphorylation of EGFR and its downstream signaling whereas the others did.

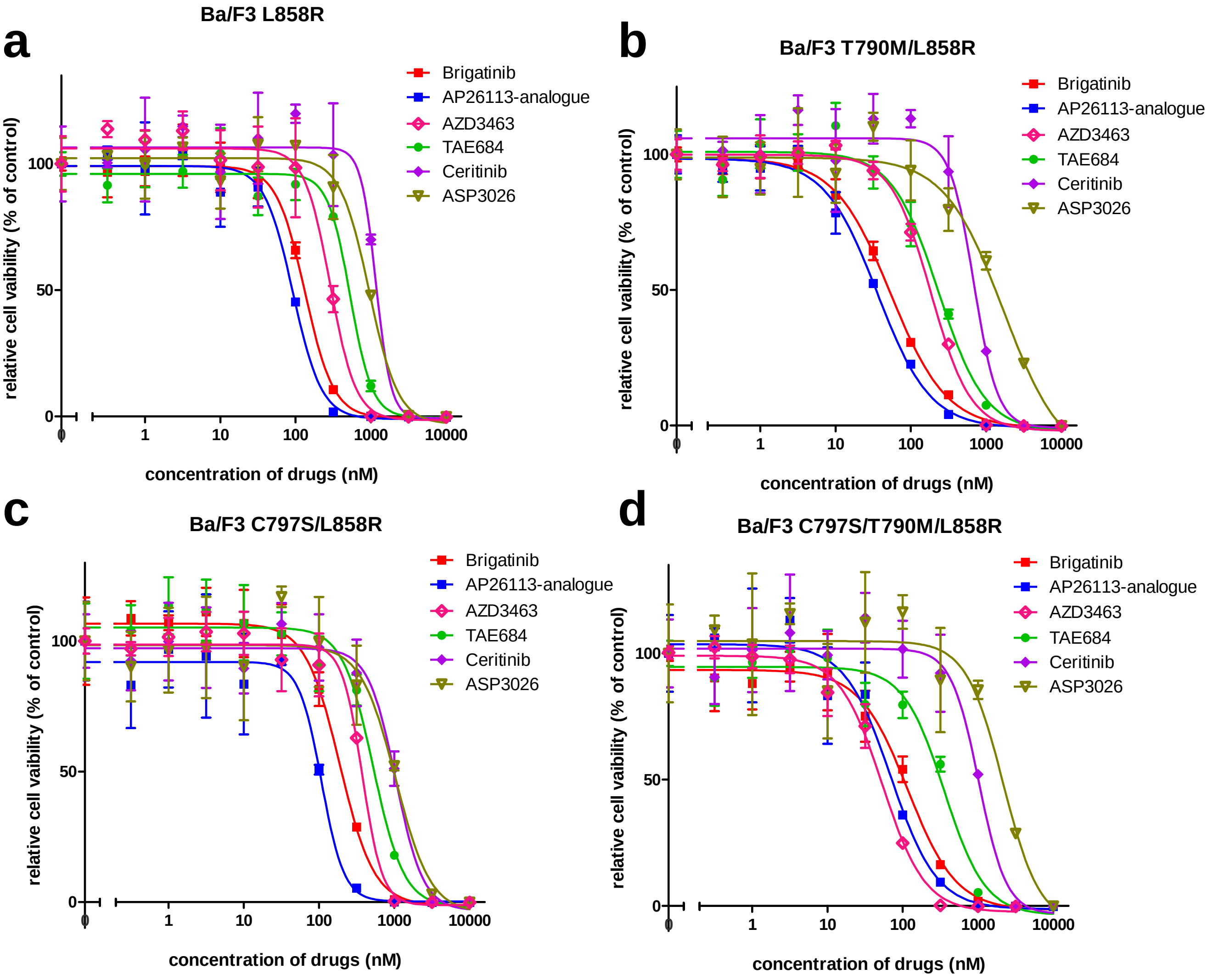

e

IC$_{50}$ (nM)	Brigatinib	AP26113-analog	AZD3463	TAE684	Ceritinib	ASP3026
L858R	132.9	92.1	287.3	528.7	1164	947.6
T790M /L858R	53.7	36.0	184.5	232.6	685.2	1691
C797S /L858R	188.3	107.9	374.5	524.1	1043	1041
C797S/T790M /L858R	116.8	69.2	167.5	355.9	1020	2148

Supplementary Figure 8: Efficacy of brigatinib and similarly structured ALK–TKIs Ba/F3 cells expressing mutant EGFR activated by L858R.
(**a**–**d**) Cell growth inhibition assessed using the CellTiter-Glo assay of EGFR-mutated Ba/F3 cells [L858R alone (**a**), T790M/L858R (**b**), C797S/L858R (**c**), C797S/T790M/L858R (**d**)] treated with brigatinib, AP26113-analog, AZD3463, TAE684, ceritinib, and ASP3026 for 72 h. ; *N*=3. Results were expressed as mean ± s.d. (**e**) Brigatinib and AP26113-analog achieved sufficiently low IC$_{50}$ values against Ba/F3 cells expressing EGFR-C797S/T790M/L858R, obtained in CellTiter-Glo assay, followed by AZD3463, although other drugs were not efficient .

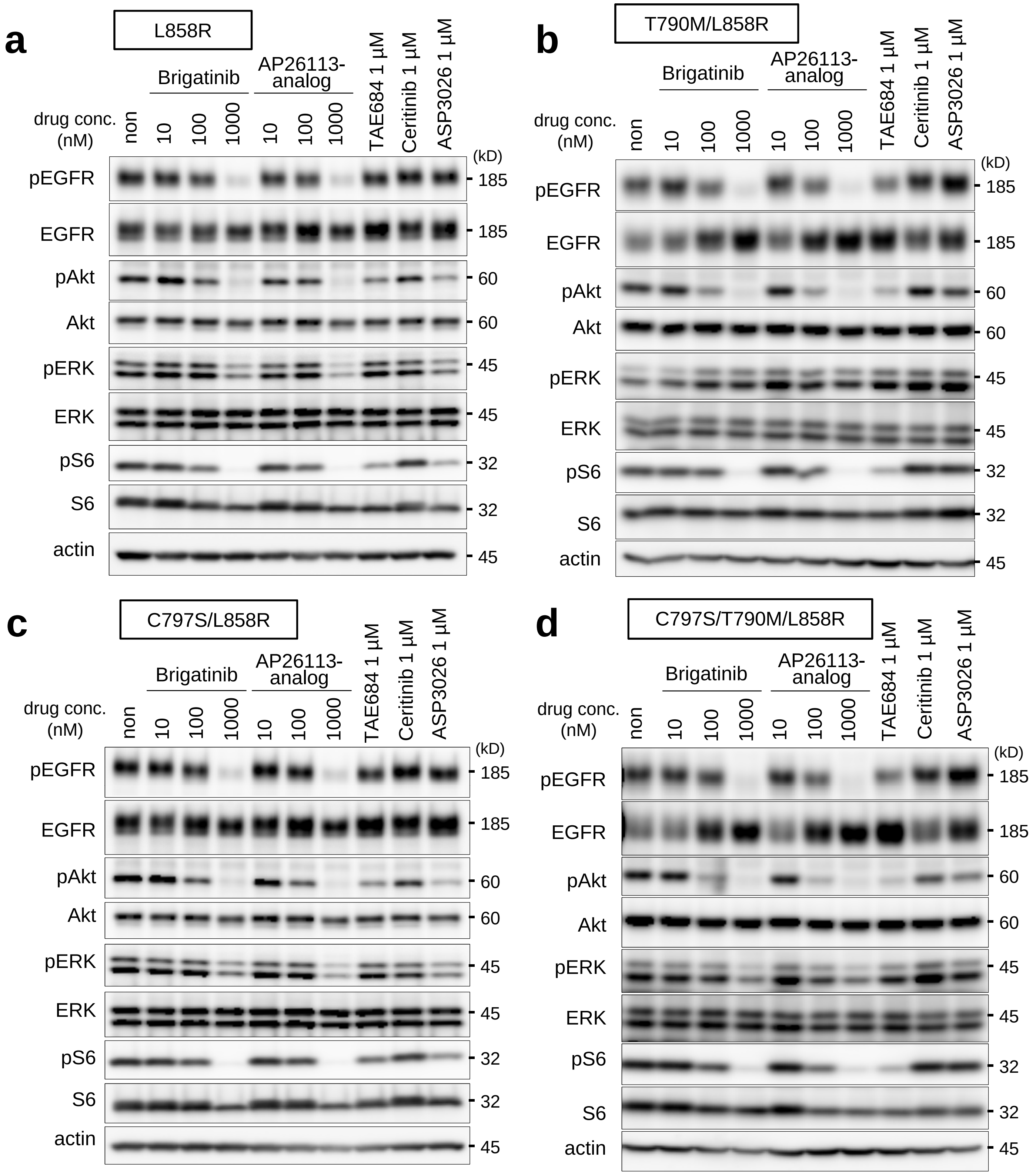

Supplementary Figure 9: Phosphorylation of EGFR and its downstream signaling in respective type of EGFR-L858R mutated Ba/F3 cells treated with brigatinib and similarly structured ALK–TKIs. (**a**-**d**) Western blotting of four types of EGFR-L858R mutated Ba/F3 [L858R (**a**), -T790M/L858R (**b**), -C797S/L858R (**c**) or -C797S/T790M/L858R (**d**)] treated with the indicated ALK–TKIs for 6 h showed that brigatinib and AP26113-analog inhibited the phosphorylation of EGFR and its downstream signal pathway in C797S/T790M/L858R, however, TAE684, ceritinib, and AP3026 didn't suppress the signals even at 1000nM.

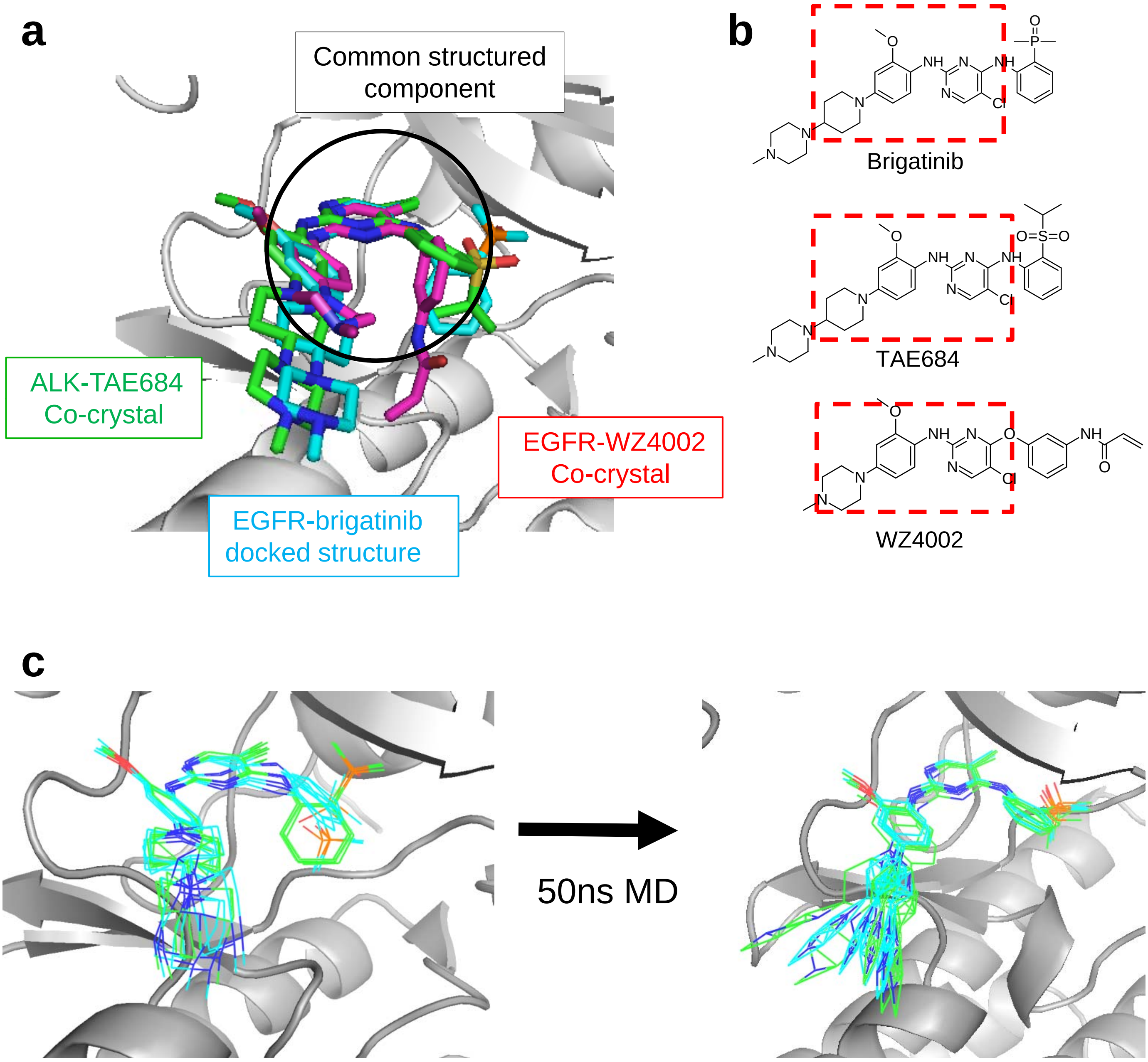

Supplementary Figure 10: Molecular docking simulation and molecular-dynamics (MD) simulation of the EGFR-C797S/T790M/L858R in complex with brigatinib.
(**a**) Molecular docking simulation indicated that brigatinib could dock into the ATP-binding site with positional restraint on the common basic structure, assuming that this substructure has a similar binding geometry between brigatinib and WZ4002. Simulation algorithm was described in the Methods. (**b**) Common structured components shown in (A) of brigatinib, TAE684 and WZ4002 were indicated by red dashed line in each chemical structure. (**c**) Ten distinctively categorized binding poses of brigatinib into the EGFR-C797S/T790M/L858R were extracted via the docking simulation from 200 candidates and used as the initial structures of the molecular-dynamics simulation (for 50 nsec). The molecular-dynamics simulation demonstrated that all ten docking modes of brigatinib in ATP-binding pocket converged into one extremely similar structure as stabilized conformation.

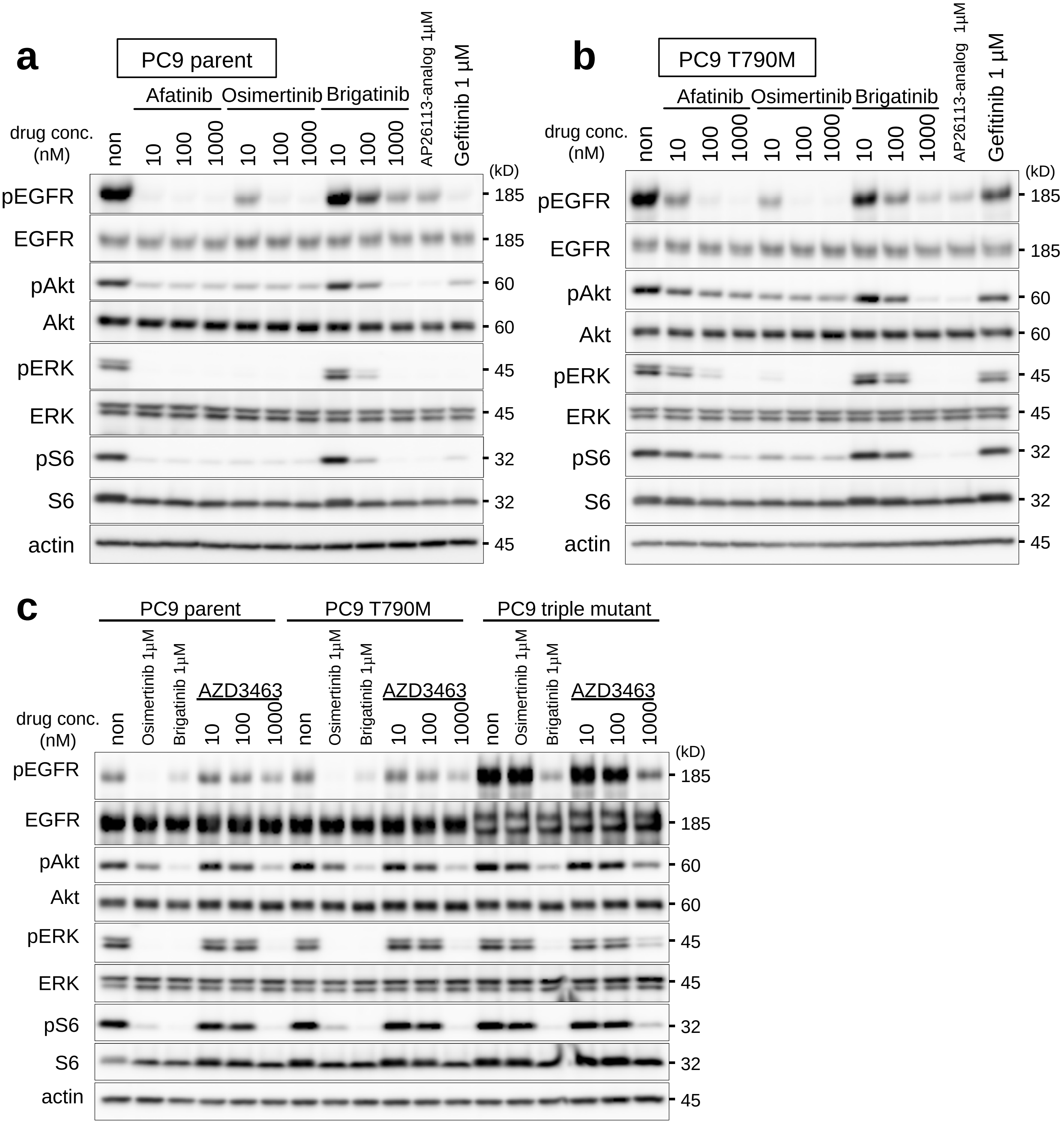

Supplementary Figure11: Signal pathway in PC9 cells treated with EGFR–TKIs, brigatinib and AZD3463.

(**a**–**b**) Phosphorylation of EGFR and its downstream signal pathway in PC9-parent (del19) (**a**) and -T790M (T790M/del19) (**b**) cells treated with indicated TKIs were evaluated using western blotting with indicated antibody. Afatinib and osimertinib showed significantly inhibit the EGFR signals of both types of PC9 cells while brigatinib and AP26113-analog moderately do.

(**c**) Western blotting of PC9 parent, -T790M, -triple mutant (C797S/T790M/del19) cells treated with osimertinib, brigatinib, and AZD3463 demonstrated that brigatinib and AZD3463 substantially achieved the inhibition of phosphorylation of EGFR and its downstream signal pathway in PC9-triple mutant cells.

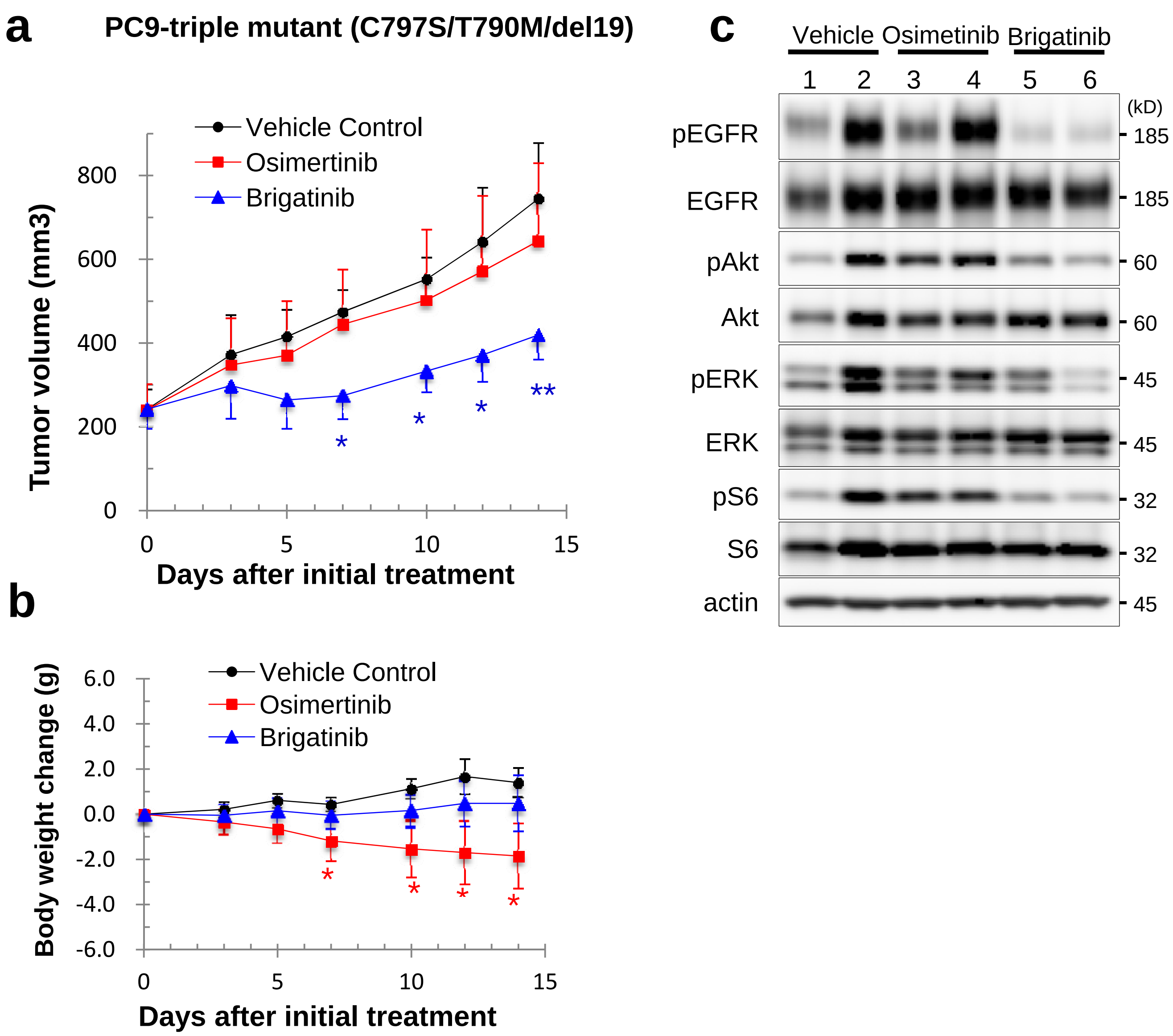

Supplementary Figure 12: Brigatinib but not osimertinib suppressed the growth of EGFR-C797S/T790M/del19 expressing PC9 cells *in vivo*.

(**a**–**b**) PC9 cells expressing EGFR-C797S/T790M/del19 were subcutaneously implanted into Balb-c nu/nu mice. When the average tumor volume reached approximately 200 mm^3, the mice were randomized into vehicle control or treatment groups (50 mg kg^{-1} of osimertinib or 75 mg kg^{-1} of brigatinib, respectively) and treated once daily by oral gavage for the indicated period. Tumor volume (V) was calculated as 0.5 × length × width^2, and body weights (B.W.) of mice were measured twice weekly. ; N=6. Results in **a** and **b** are expressed as mean ± s.d. ; **P < 0.01, *P < 0.05 (Mann–Whitney U test).

(**c**) Phosphorylation of EGFR and its downstream signaling in two tumor samples obtained from each group were evaluated using western blotting.

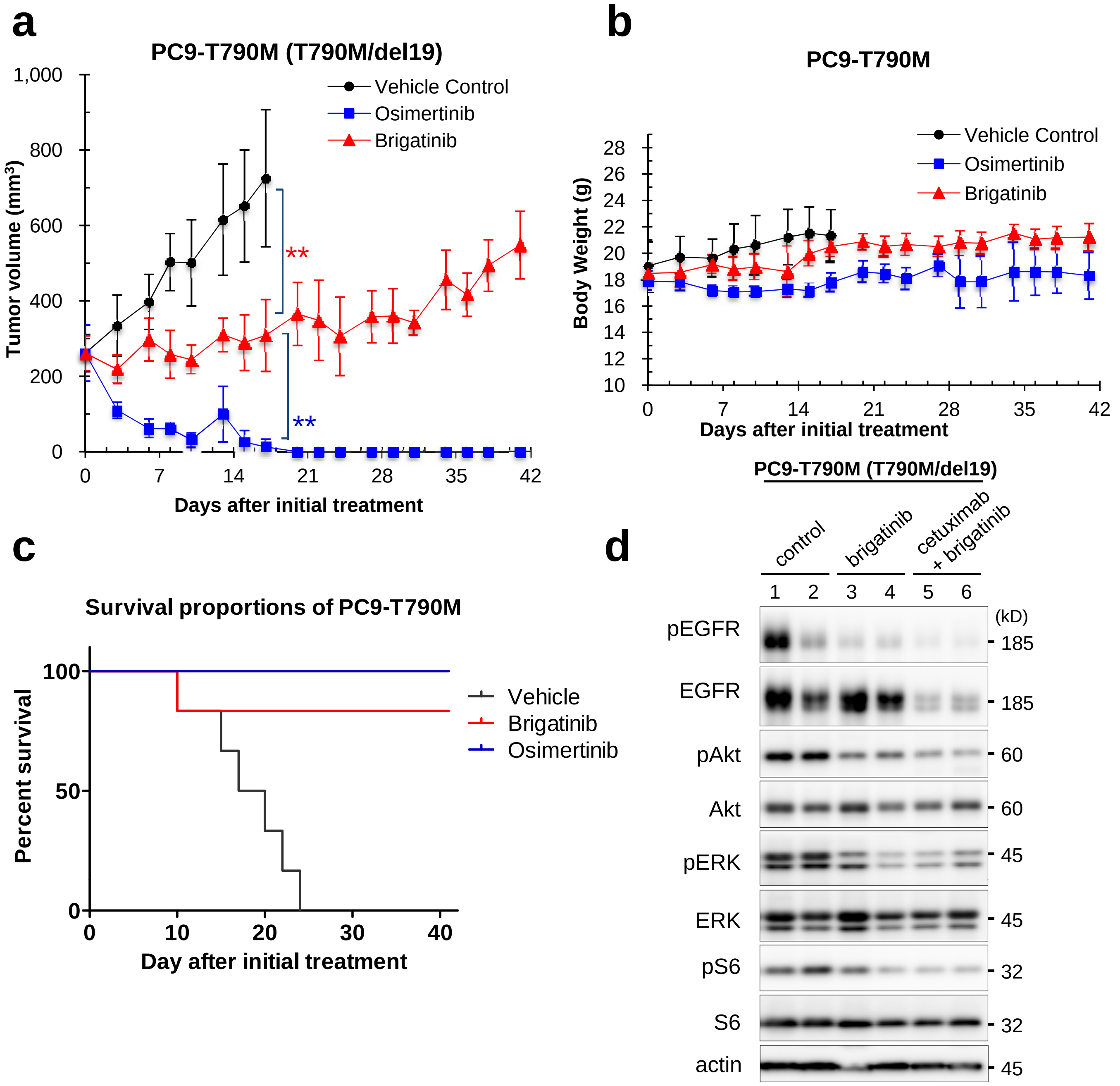

Supplementary Figure 13: Brigatinib with cetuximab inhibited the tumor growth of EGFR-T790M/exon19 deletion mutation harboring PC9 cells in vivo.
(**a**–**b**) Gefitinib resistant PC9 cells expressing EGFR-T790M/del19 (PC9-T790M) were subcutaneously implanted into Balb-c nu/nu mice. When the average tumor volume reached approximately 200 mm^3, the mice were randomized into vehicle control or treatment groups (50 mg kg^{-1} of osimertinib or 75 mg kg^{-1} of brigatinib, respectively) and treated once daily by oral gavage for the indicated period. Tumor volume (V) was calculated as 0.5 × length × $width^2$, and body weights (B.W.) of mice were measured twice weekly. ; N=6. Results in **a** and **b** are expressed as mean ± s.d. ; $^{**}P < 0.01$ (Mann–Whitney U test).(**c**) Survival proportion described using the Kaplan–Meier method showed osimertinib and brigatinib prolonged the survival of mice into whom PC9-T790M xenogfrafts were implanted. One brigatinib treated mice was accidentally killed by the procedure of oral gavage at day 10. (**d**) Balb-c nu/nu mice bearing the PC9-T790M cells treated with vehicle control or treatment groups (75 mg kg^{-1} of brigatinib or 75 mg kg^{-1} of brigatinib with 1 mg per mouse of cetuximab, respectively). Brigatinib was treated once daily by oral gavage, and cetuximab was treated 3 times/week. After 10 days of the treatment, phosphorylation of EGFR and its downstream signaling in two tumor samples obtained from each group were evaluated using western blotting.

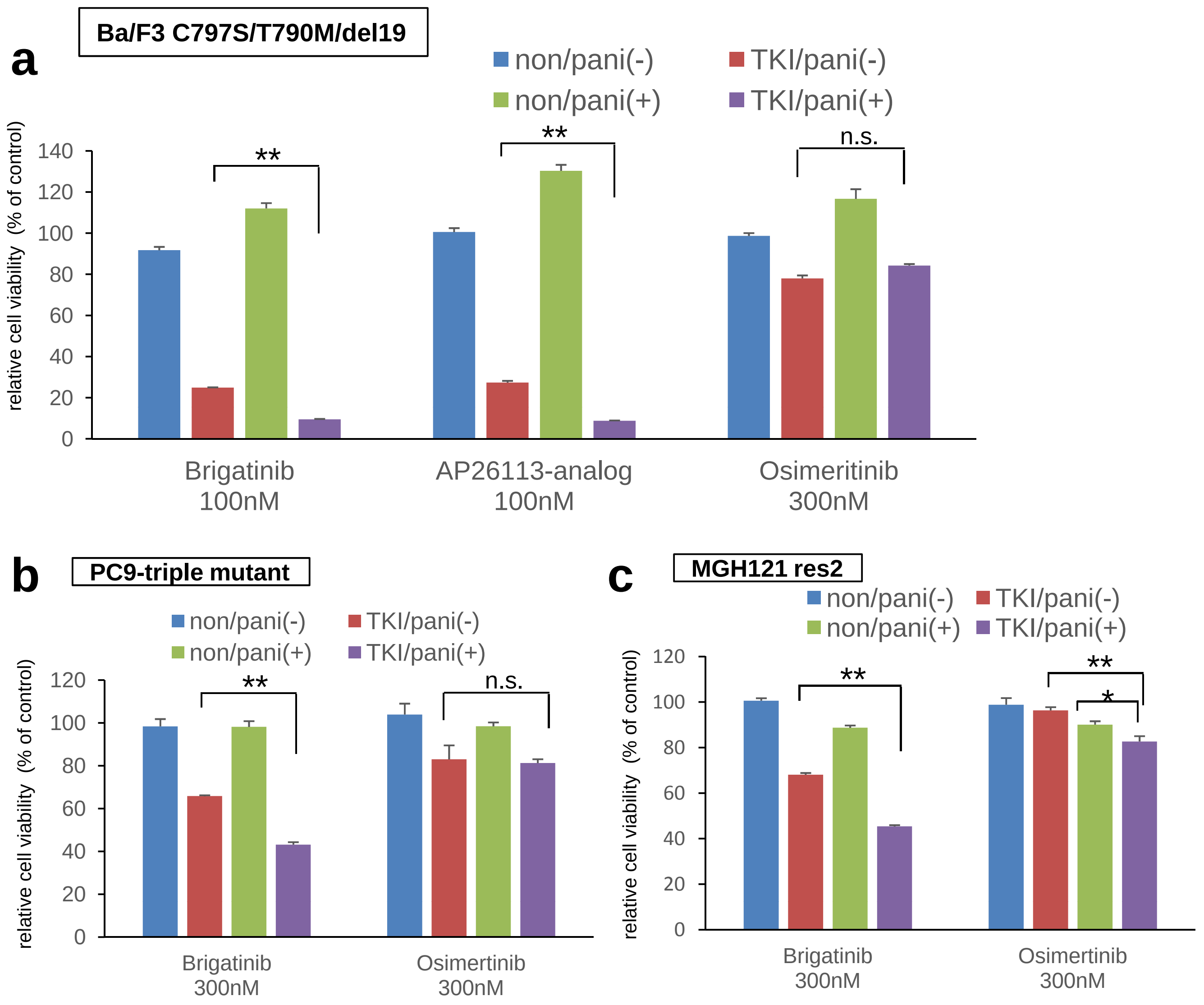

Supplementary Figure 14: Combination with panitumumab enhanced the potency of brigatinib
(**a**) The cell growth inhibition of Ba/F3 cells expressing EGFR-C797S/T790M/del19 (EGFR-triple-del19) treated with brigatinib, AP26113-analog and osimertinib at indicated concentrations combined with or without panitumumab (20 µg/ml) for 72 h assessed using CellTiter-Glo assay. (**b**–**c**) The cell growth inhibition of PC9-triple mutant cells (**b**) or MGH121 res2 cells (**c**), both expressing EGFR-triple-del19, treated with brigatinib and osimertinib at indicated concentrations combined with or without panitumumab (20 µg/ml) for 72 h assessed using CellTiter-Glo assay. Results are expressed as mean ± s.d. *N*=3. ; n.s.: not significant, ***P* < 0.01, **P* < 0.05 (Student's *t*-test).

Supplementary Figure 15: Original immunoblots for indicated figures

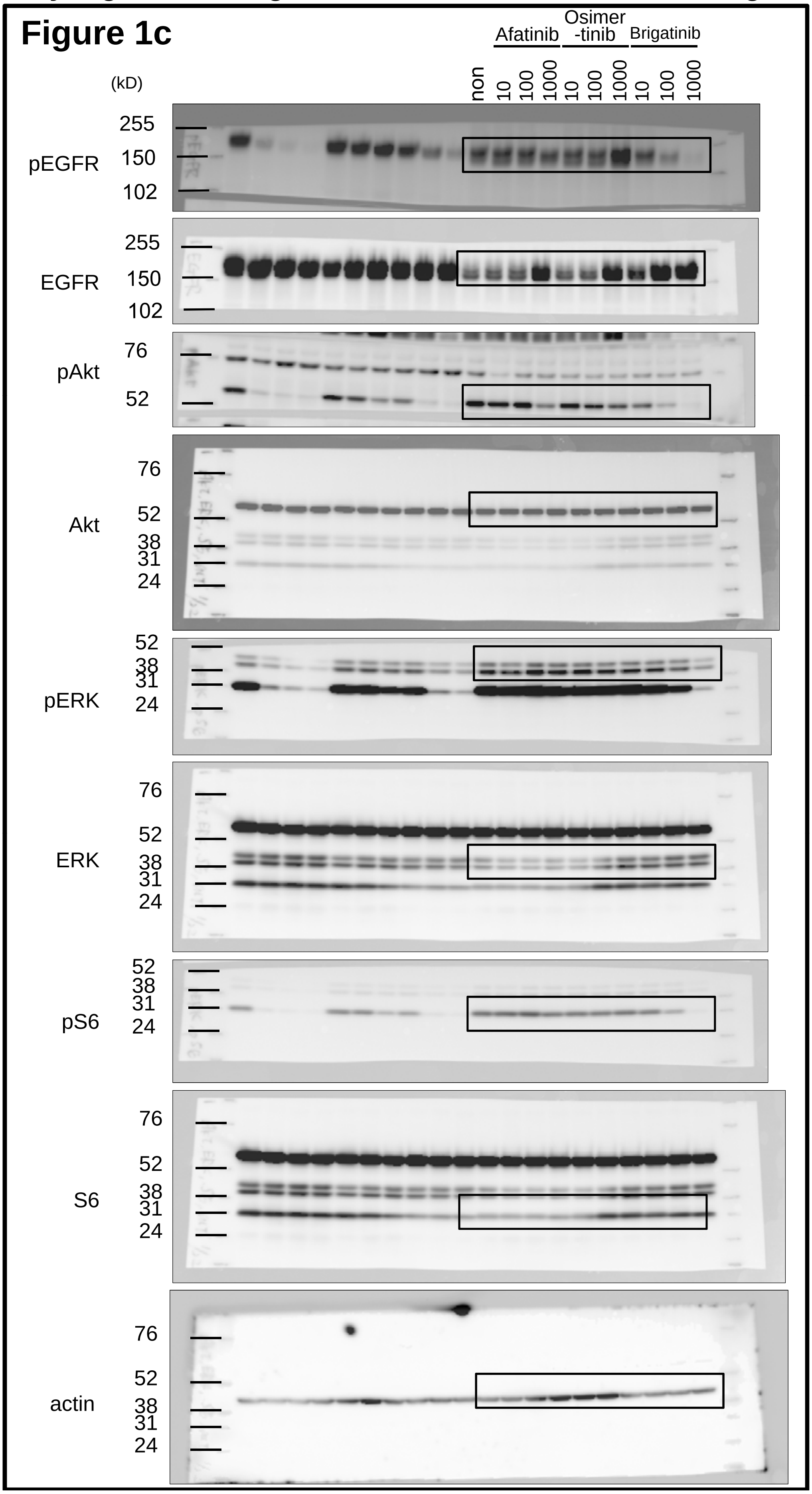

Supplementary Figure 16: Original immunoblots for indicated figures

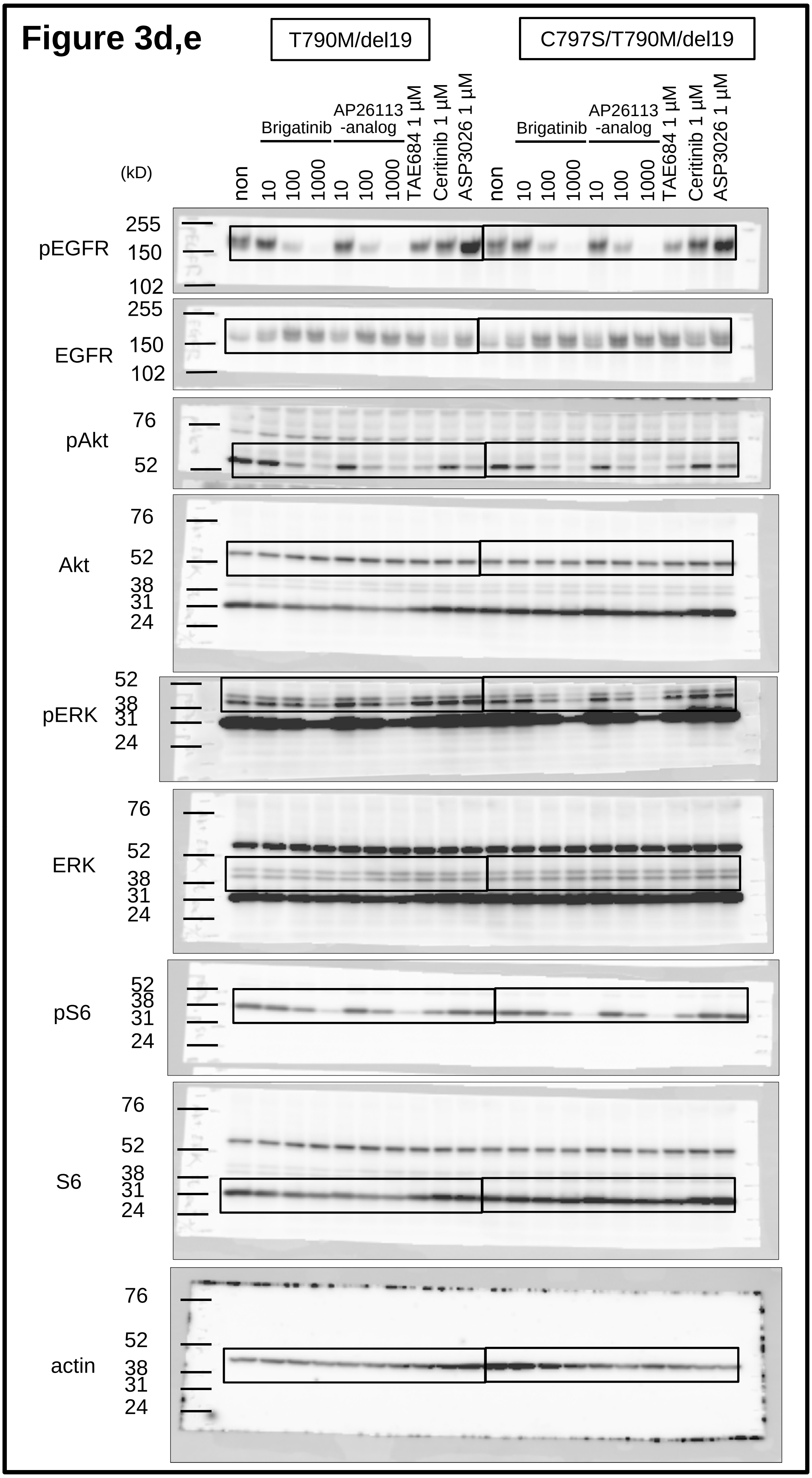

Supplementary Figure 17: Original immunoblots for indicated figures

Figure 5f

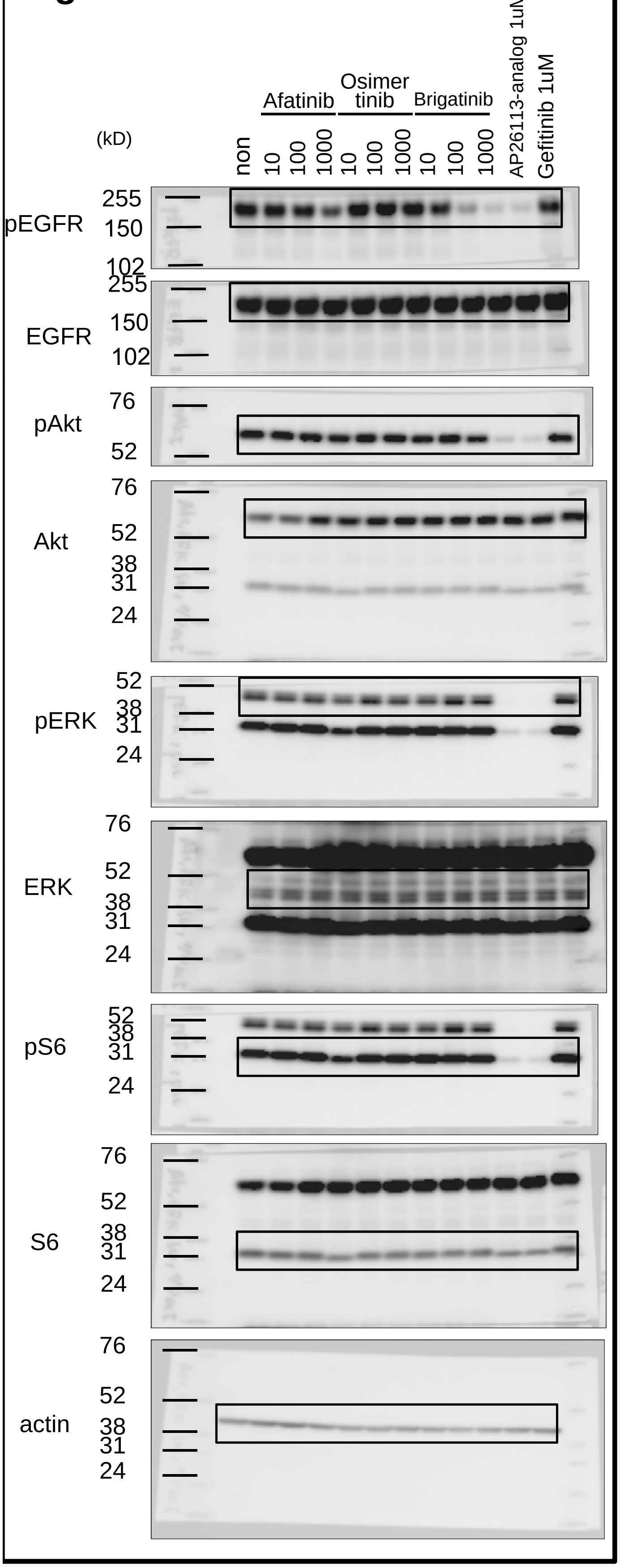

Figure 5g

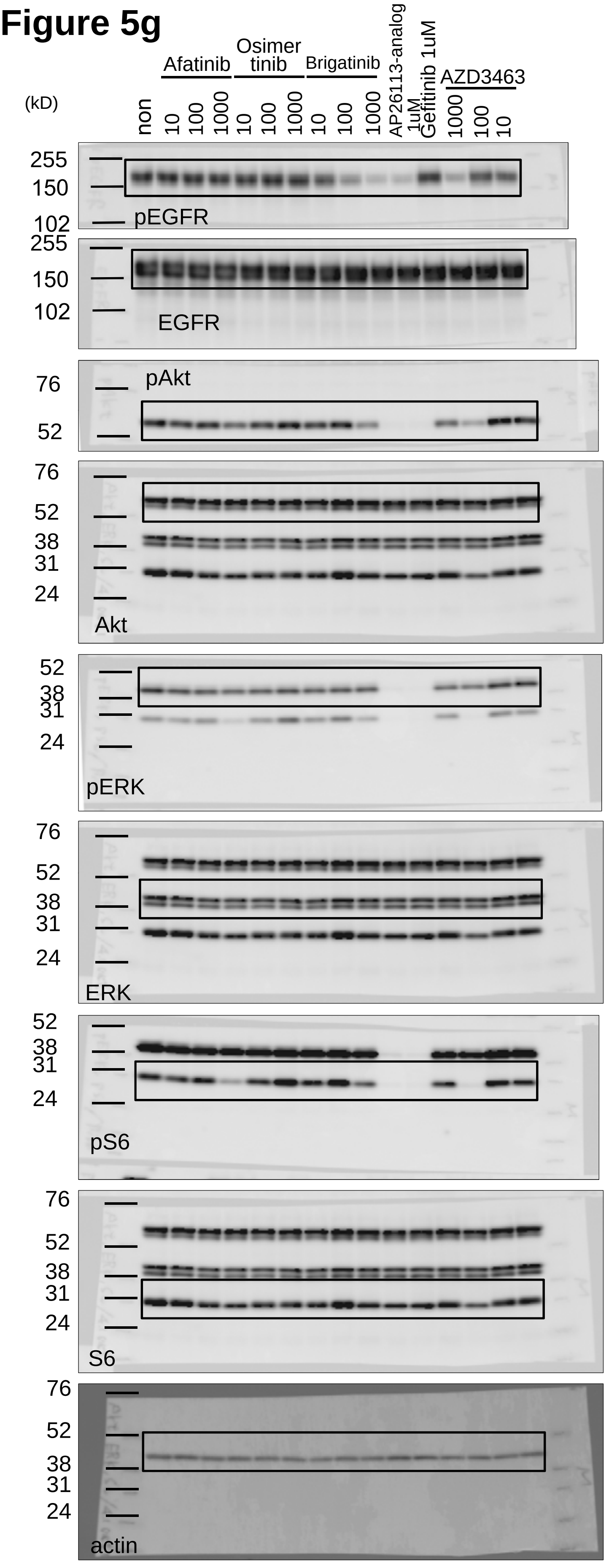

Supplementary Figure 18: Original immunoblots for indicated figures

Figure 6b

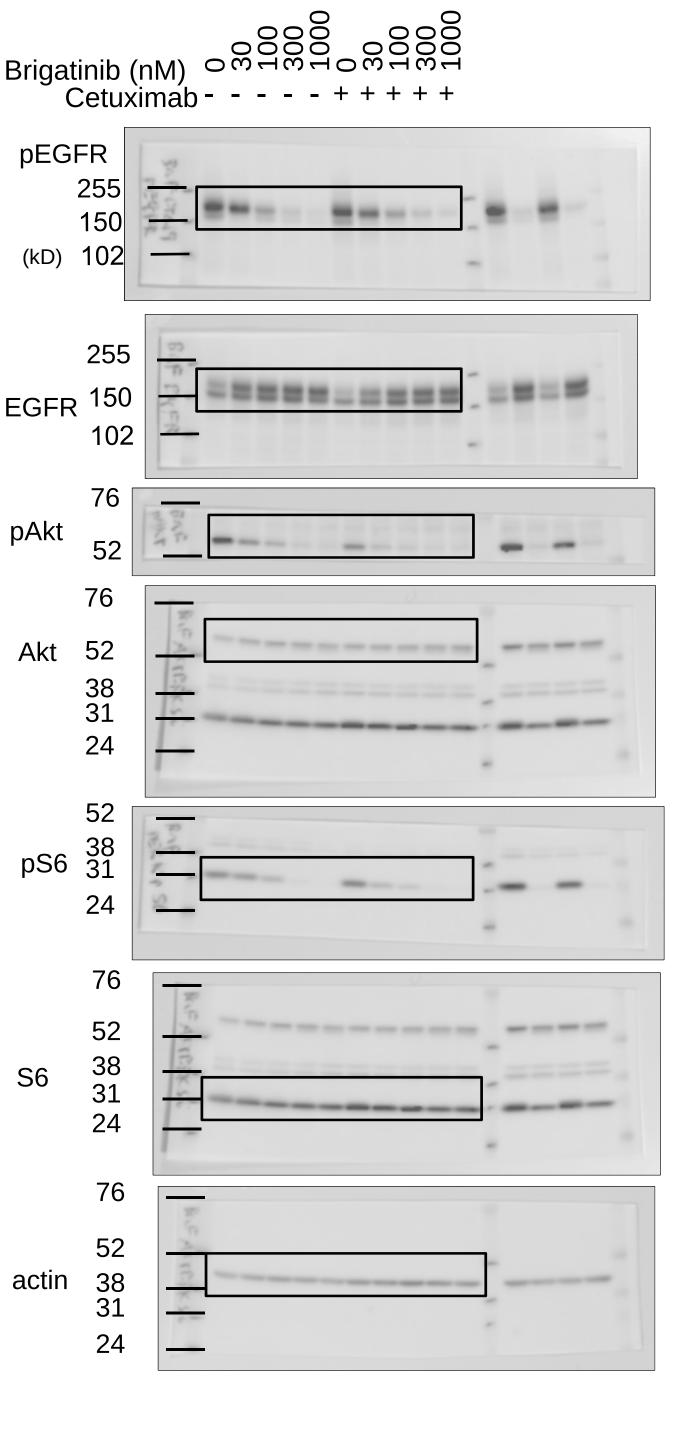

Figure 6d

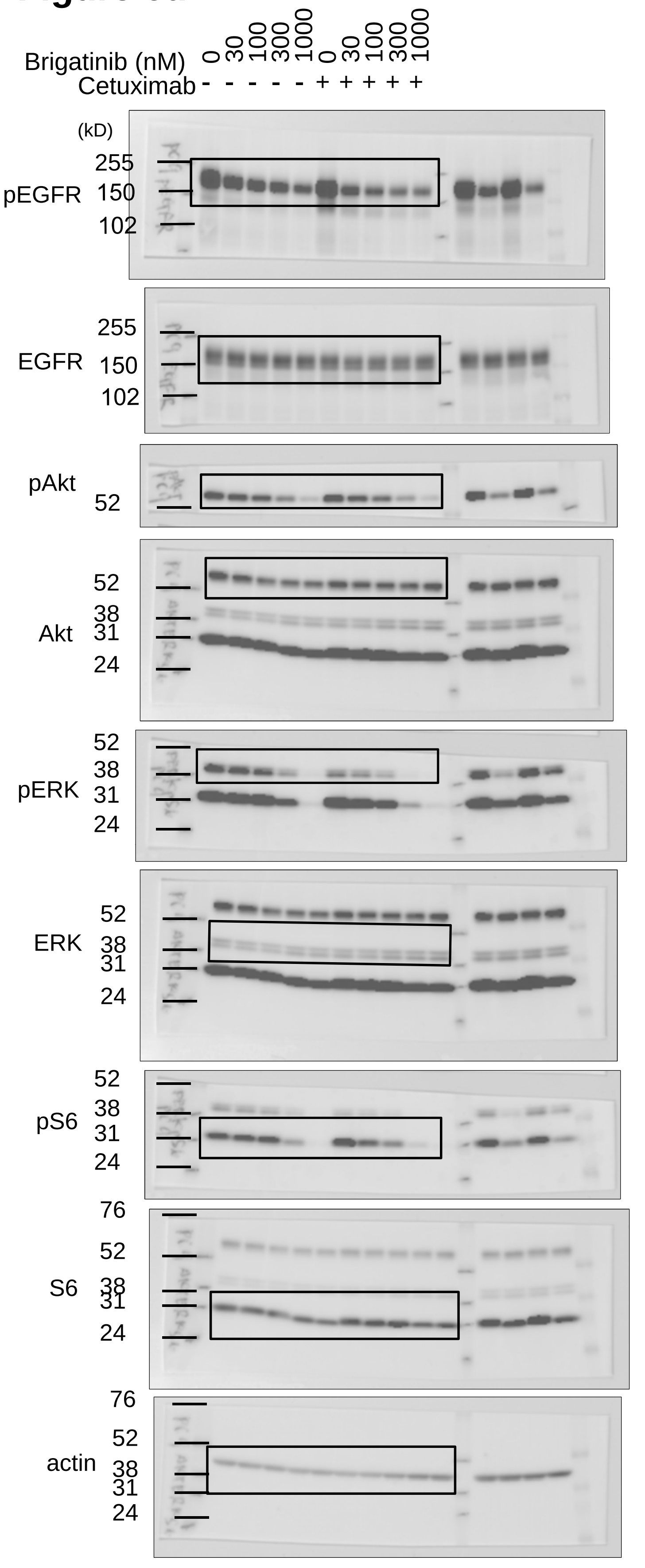

Supplementary Figure 19: Original immunoblots for indicated figures

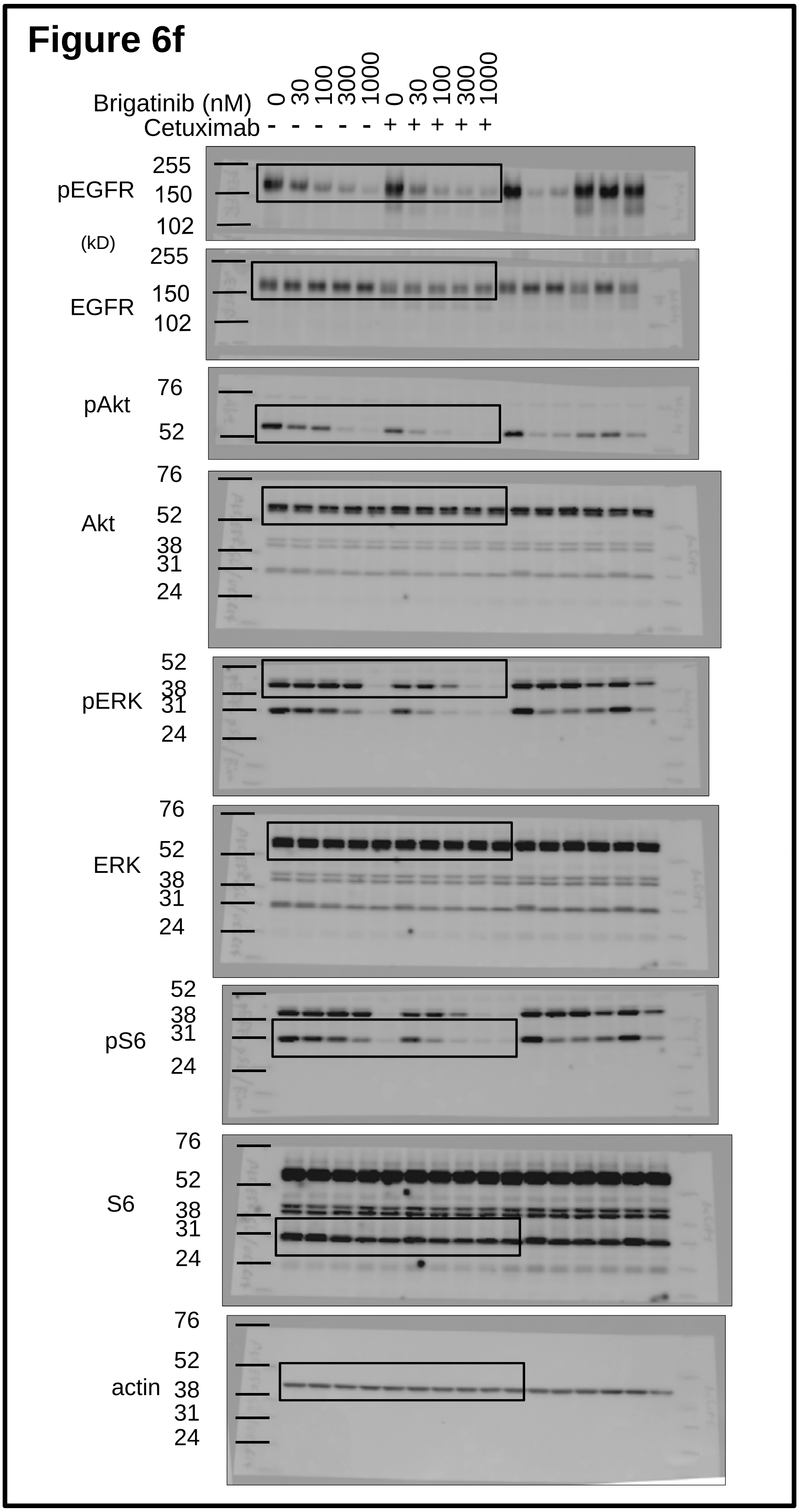

Supplementary Figure 20: Original immunoblots for indicated figures

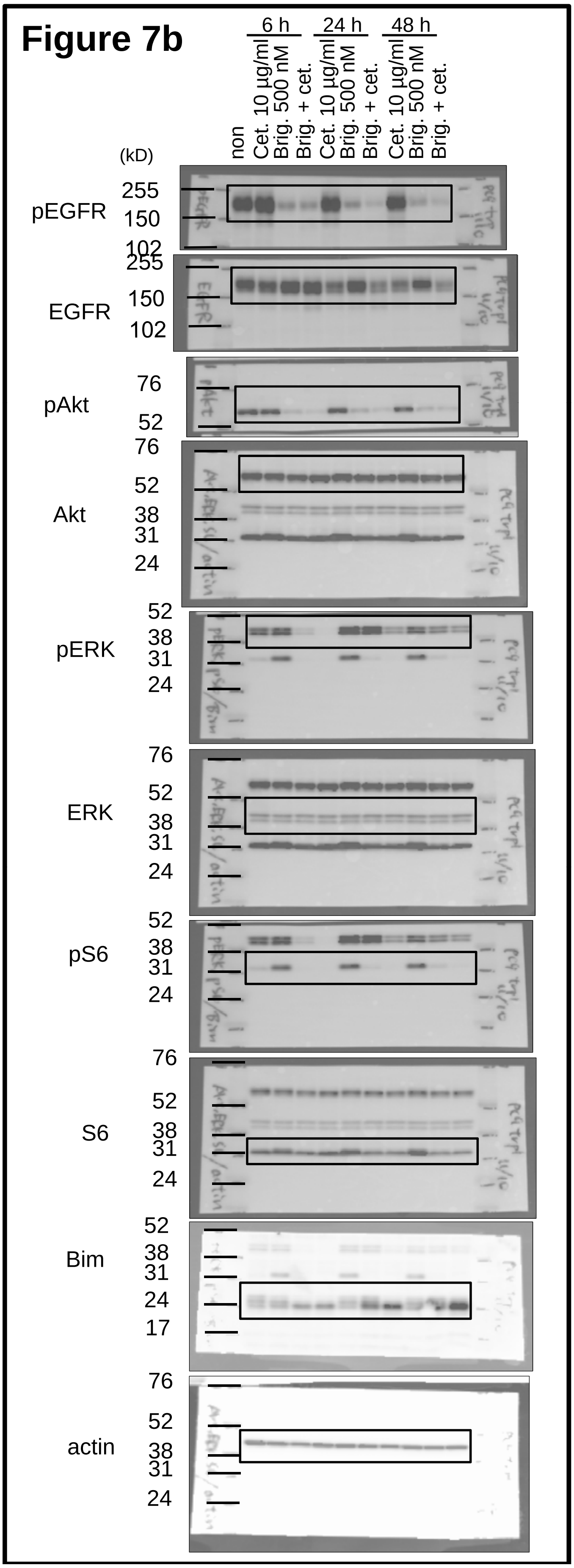

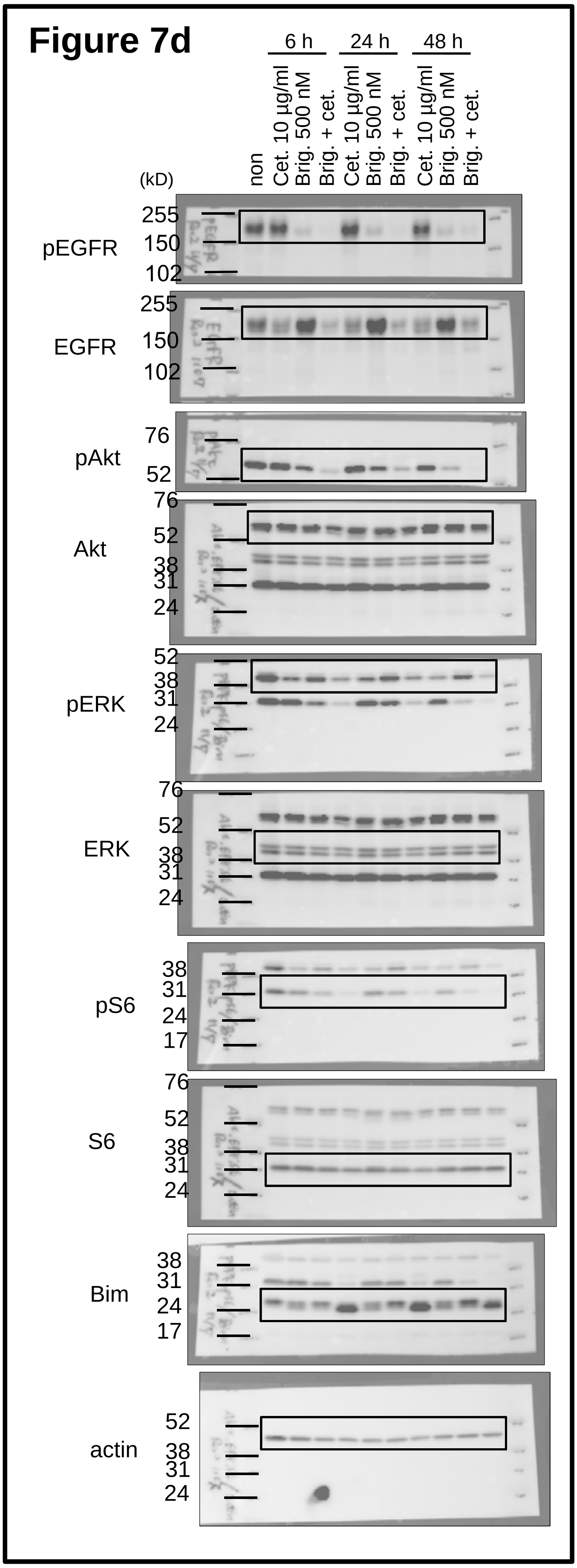

Supplementary Figure 21: Original immunoblots for indicated figures

Figure 8d

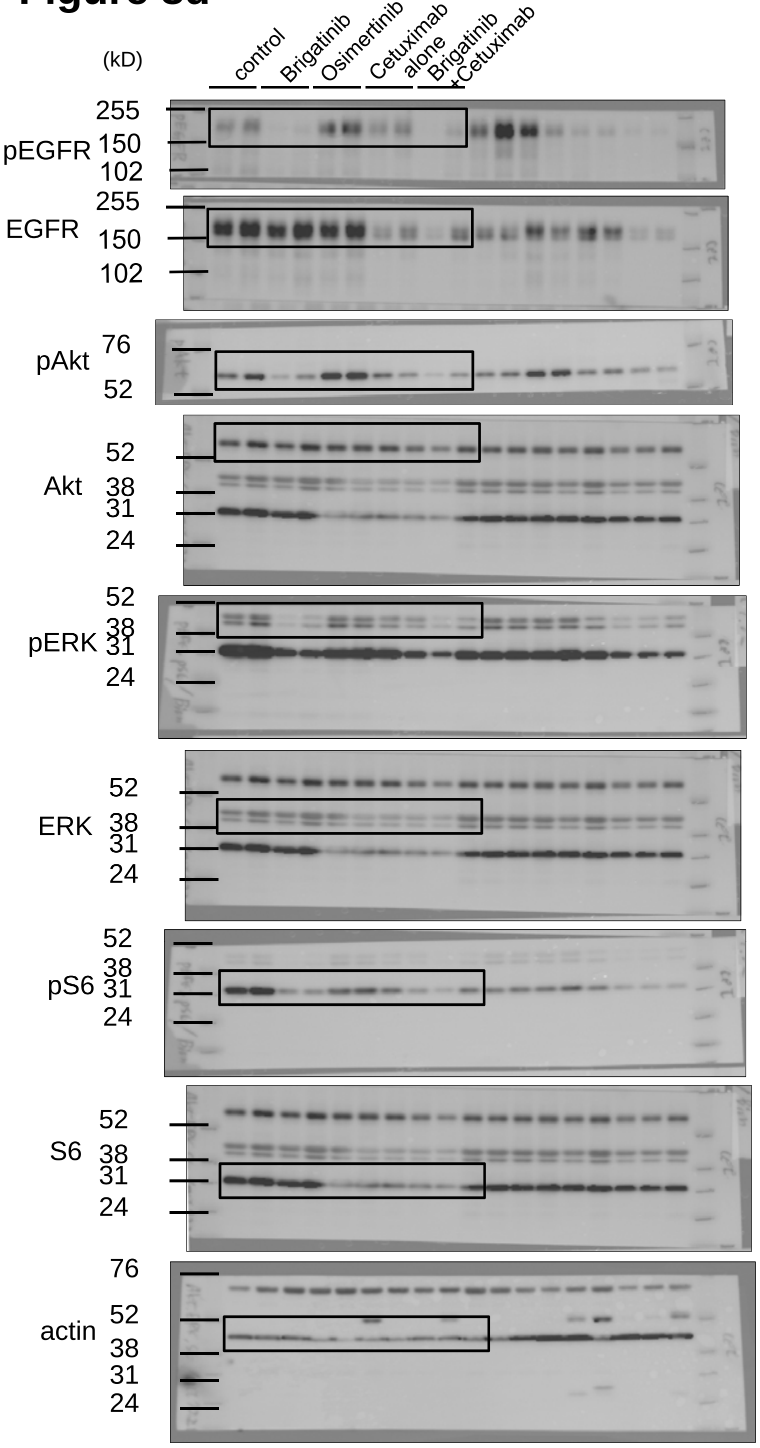

Figure 8h

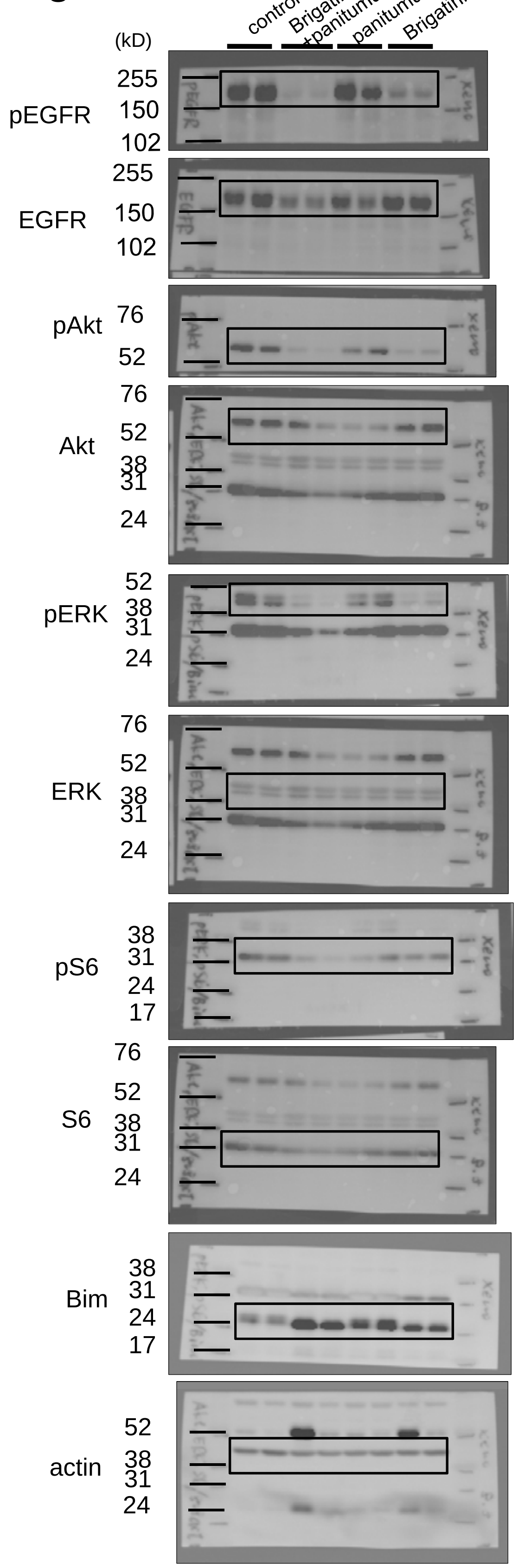

	Drug	Company
1	17-AAG	LC laboratories
2	AEW541	ActiveBiochem
3	Afatinib	ChemieTek
4	Alectinib	ActiveBiochem
5	AP26113-analogue	Selleck
6	ASP3026	ChemieTek
7	AZD3463	BioVision
8	BGJ398	Shanghai Biochem
9	Brigatinib	Shanghai Biochem
10	Cabozantinib	ActiveBiochem
11	CEP701	Calbiochem
12	Ceritinib	ActiveBiochem
13	CO-1686	ActiveBiochem
14	Crizotinib	Biochempartner
15	E7080	Selleck
16	Cetuximab	Merck

	Drug	Company
17	EGF-816	ChemScene
18	Erlotinib	LC laboratories
19	Foretinib	AdooQ Bioscience
20	Gefitinib	LC laboratories
21	Imatinib	LC laboratories
22	Lapatinib	LC laboratories
23	Lorlatinib	ActiveBiochem
24	Nintedanib	Selleck
25	Osimertinib	Selleck
26	PHA665752	Tocris Bioscience
27	Ponatinib	Selleck
28	Sorafenib	Selleck
29	Sunitinib	Selleck
30	TAE684	ChemieTek
31	Vandetanib	Shanghai Biochem
32	Panitumumab	Takeda Pharma.

Supplementary Table 1: Kinase inhibitors and other drugs

The drugs used in the experiments and the companies from which they were purchased are shown in table.
