# Supplementary material for: Brigatinib combined with anti-EGFR antibody overcomes osimertinib resistance in EGFR-mutated non-small-cell lung cancer
Source: Nat Commun. 2017 Mar 13;8:14768. doi: 10.1038/ncomms14768 (PMC5355811; doi:10.1038/ncomms14768)
Supplement: Peer Review File [file ncomms14768-s3.pdf]

Editorial Note: this manuscript has been previously reviewed at another journal that is not operating a transparent peer review scheme. This document only contains reviewer comments and rebuttal letters for versions considered at Nature Communications.

REVIEWERS' COMMENTS:

Reviewer #2 (Remarks to the Author):

The authors have addressed all of my comments.

REVIEWERS' COMMENTS:

Reviewer #2 (Remarks to the Author):

The authors have addressed all of my comments.

Answer ==> Thank you very much for reviewing our manuscript and thank you so much for your kind decision
